# Supplementary figures and images for: Duckweed hosts a taxonomically similar bacterial assemblage as the terrestrial leaf microbiome
Source: PLoS One. 2020 Feb 6;15(2):e0228560. doi: 10.1371/journal.pone.0228560 (PMC7004381; doi:10.1371/journal.pone.0228560)

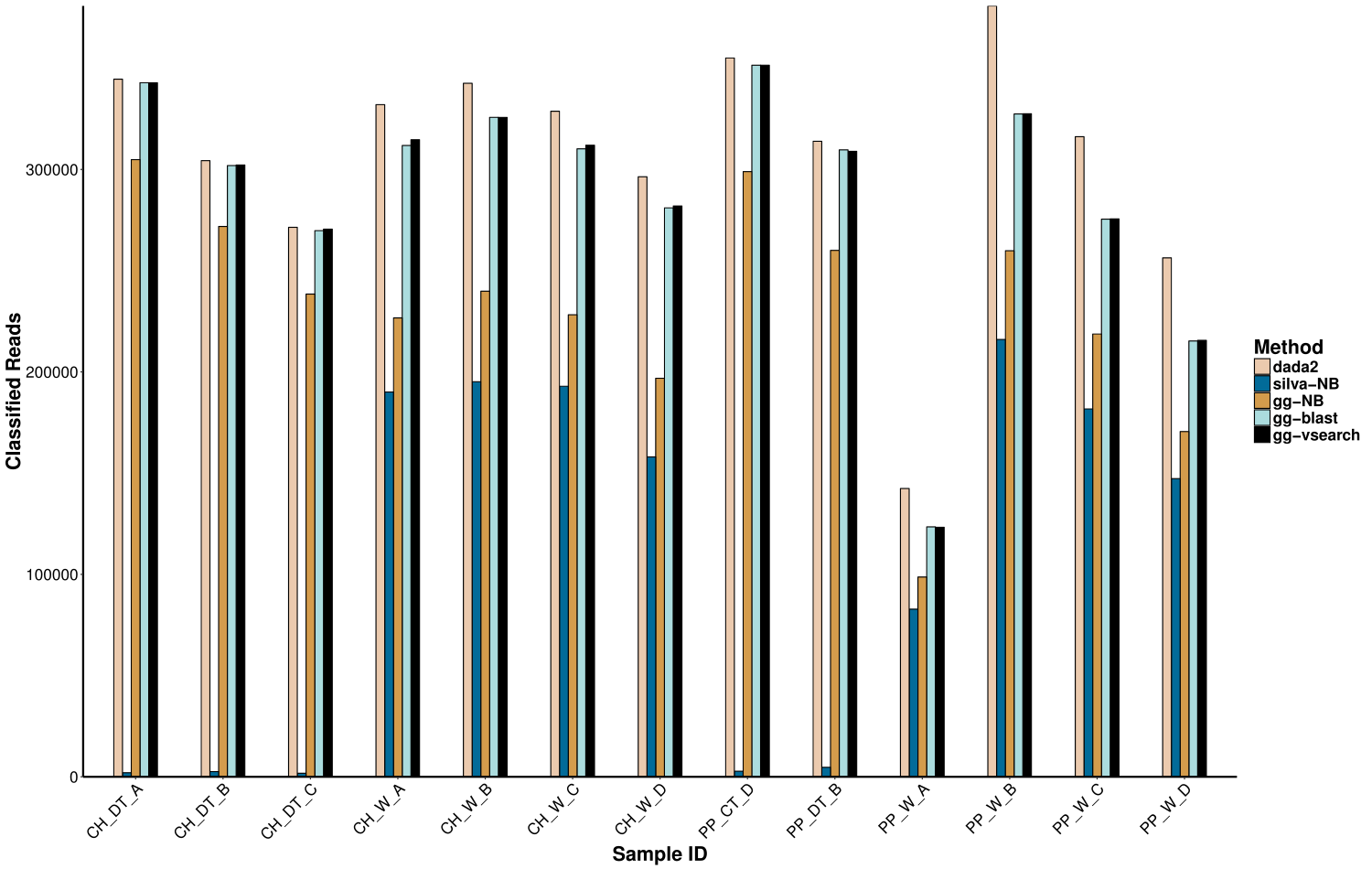

Supplement: S1 Fig — Different methods for the q2 feature-classifier plugin were tested for their ability to classify ASVs at the genus level. The effect of using different databases was tested by either using the Greengenes 13_8 99% OTUs reference database (gg) or SILVA 132 (silva) database. Default parameters were used in each instance. dada2 = reads remaining after q2-dada2 quality control, NB = q2 feature classifier using naive bayes method, blast = q2 feature classifier using BLAST+ consensus method, vsearch = q2 feature-classifier using VSEARCH consensus method. (TIF) [file pone.0228560.s001.tif]

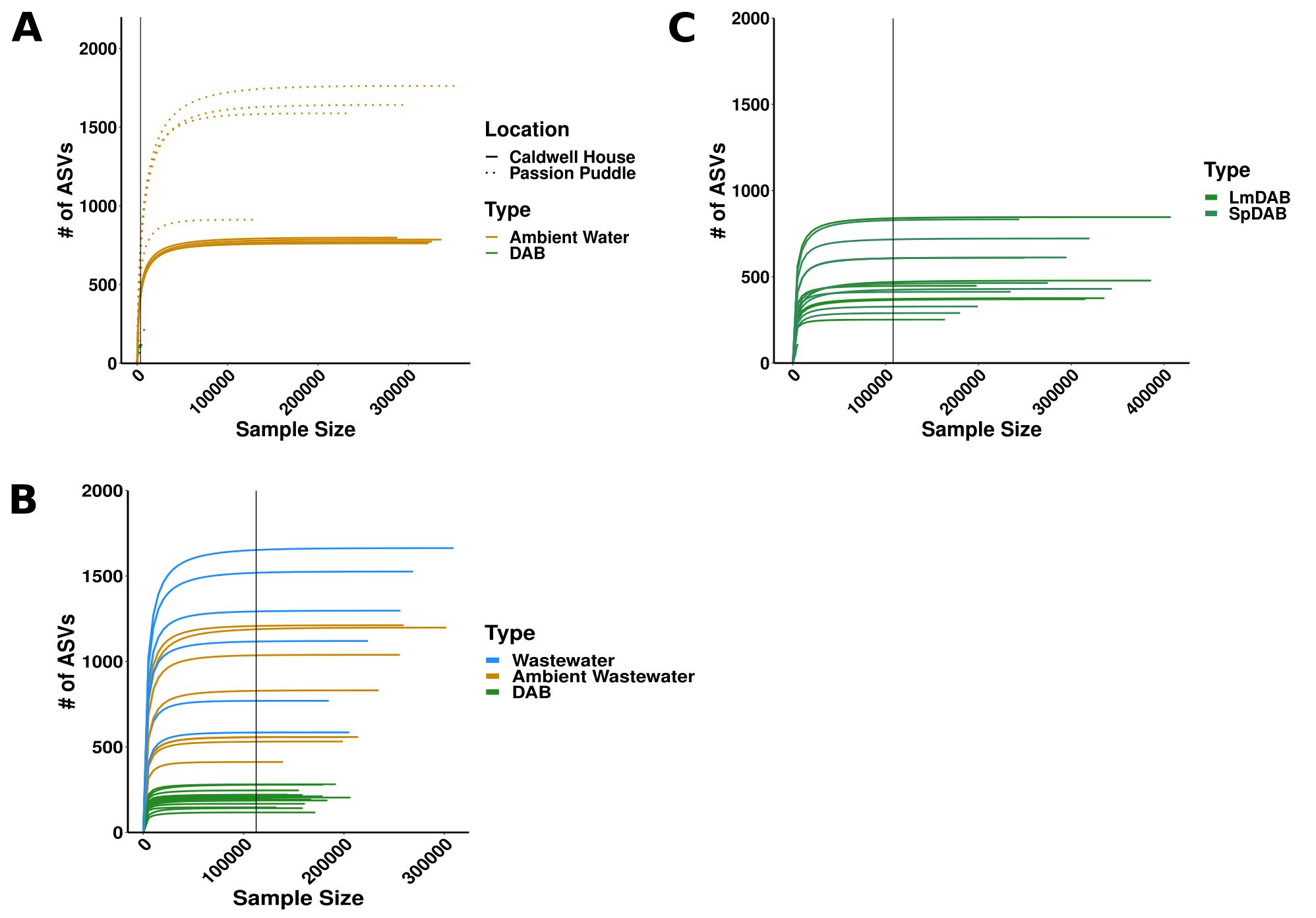

Supplement: S2 Fig — (A) The number of ASVs observed in Caldwell House and Passion Puddle bacterial communities at different sampling depths. The solid line intercepting the x-axis represents a sampling depth of 3664 reads. (B) The number of ASVs observed in Princeton Meadows year 1 bacterial communities at different sampling depths. Samples were rarefied to 112500 reads. (C) The number of ASVs observed in Princeton Meadows year 2 bacterial communities at different sampling depths. Samples were rarefied to 108000 reads. (TIF) [file pone.0228560.s002.tif]

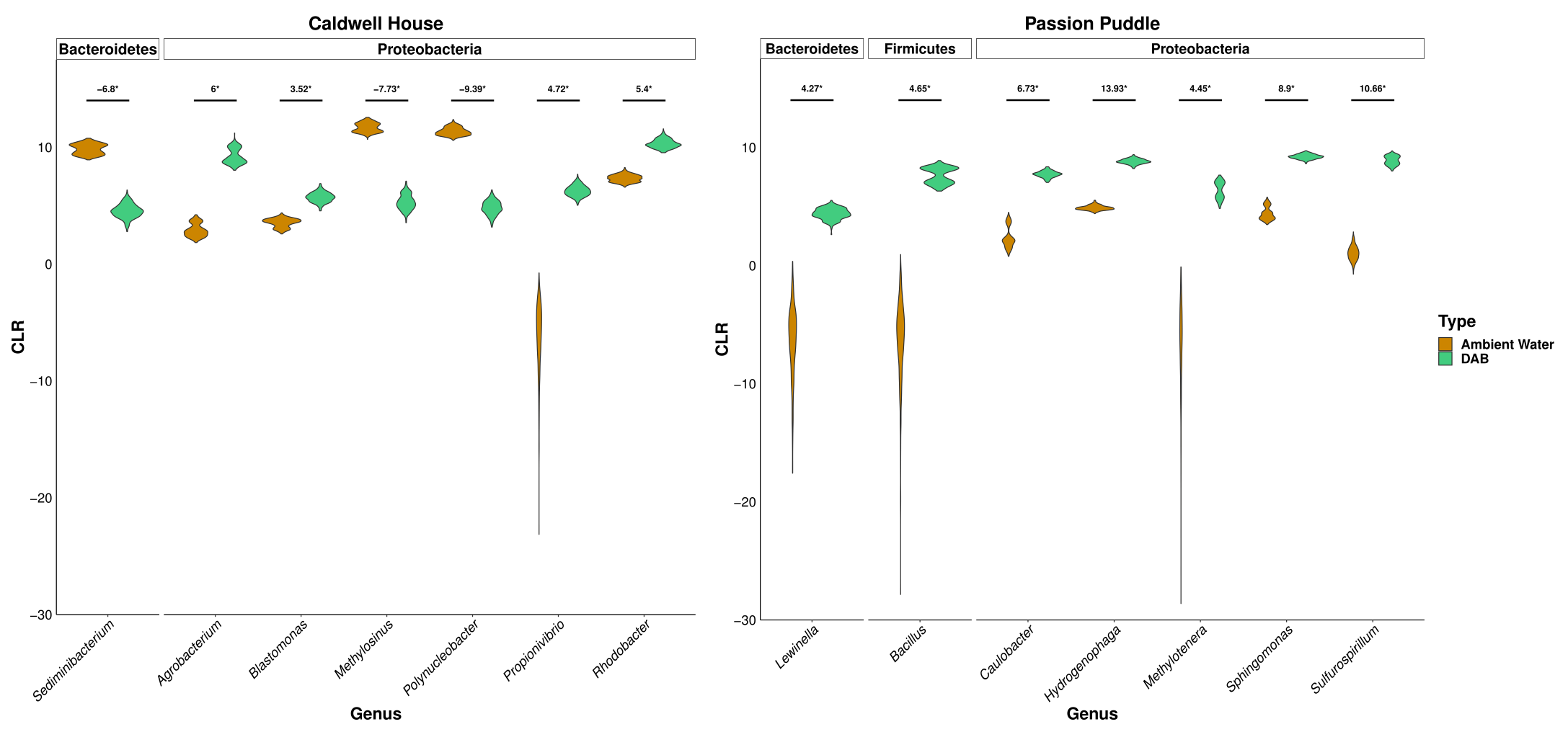

Supplement: S3 Fig — ALDEx2 was performed to determine bacterial genera whose abundance was significantly different between duckweed-associated bacterial (DAB) community and ambient water communities from Caldwell House and Passion Puddle. Violin plots display the distribution of centered-log ratios (CLR) for bacterial genera whose abundance was significantly different between communities (“*” = adjusted Welch’s t-test, p-value < 0.05). Effect sizes are displayed for each bacterial genus. Larger values signify a greater difference between communities. Positive effect sizes represent a higher abundance in DAB community compared to ambient water community while negative effect sizes represent a higher abundance in ambient water community compared to DAB community. (TIF) [file pone.0228560.s003.tif]

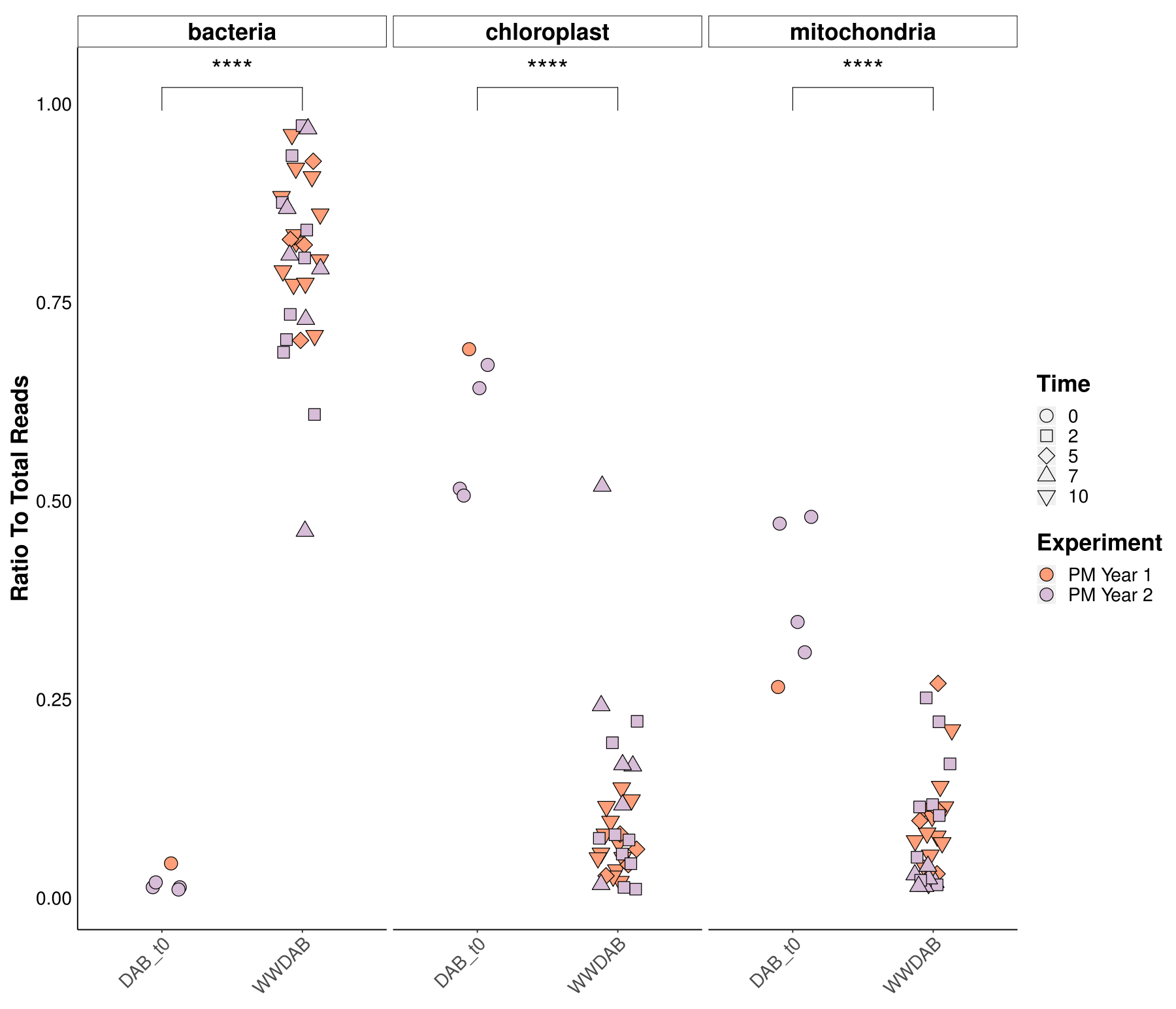

Supplement: S4 Fig — Number of plastid and bacteria reads normalized to total reads in initial quasi-gnotobiotic Sp9509 (DAB t0) compared to Sp9509 duckweed tissue several days after inoculation with wastewater from both Princeton Meadows year 1 and year 2 studies (WWDAB). Pairwise comparison was performed using Wilcoxon rank sum test with p-values displayed. (TIF) [file pone.0228560.s004.tif]

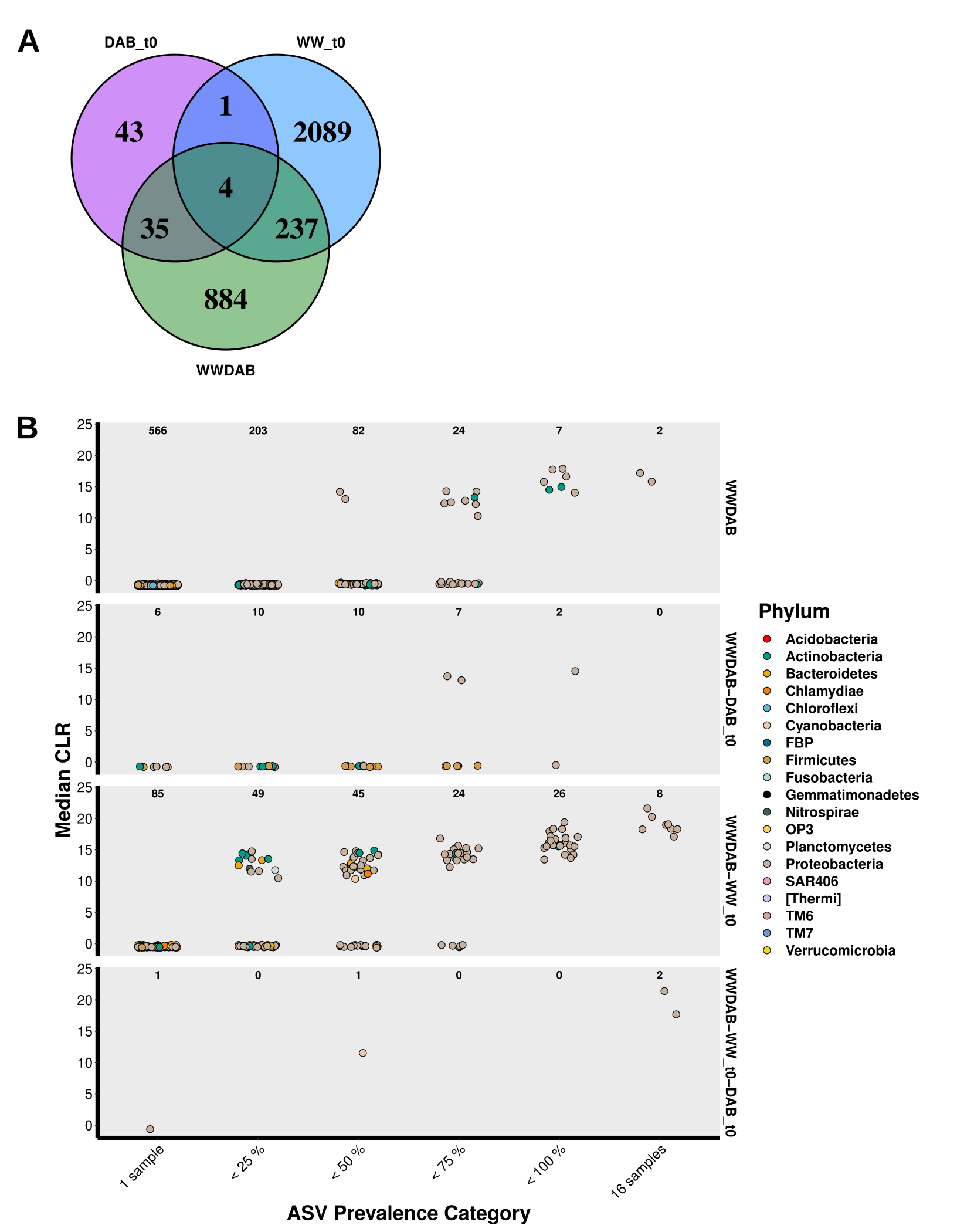

Supplement: S5 Fig — (A) Venn diagram showing the number of bacterial ASVs specific to and shared between the initial Sp9509 tissue (DAB _t0), initial wastewater inoculum (WW_t0), and Sp9509 inoculated with wastewater (WWDAB). (B) For each bacterial ASV found in the WWDAB community (n = 16), we calculated abundance (median clr), the amount of samples the ASV was found in (ASV Prevalence Category), and the communities the ASV was found in (WWDAB, WW_t0, DAB_t0). Each data point in the graph represents a bacterial ASV. (TIF) [file pone.0228560.s005.tif]

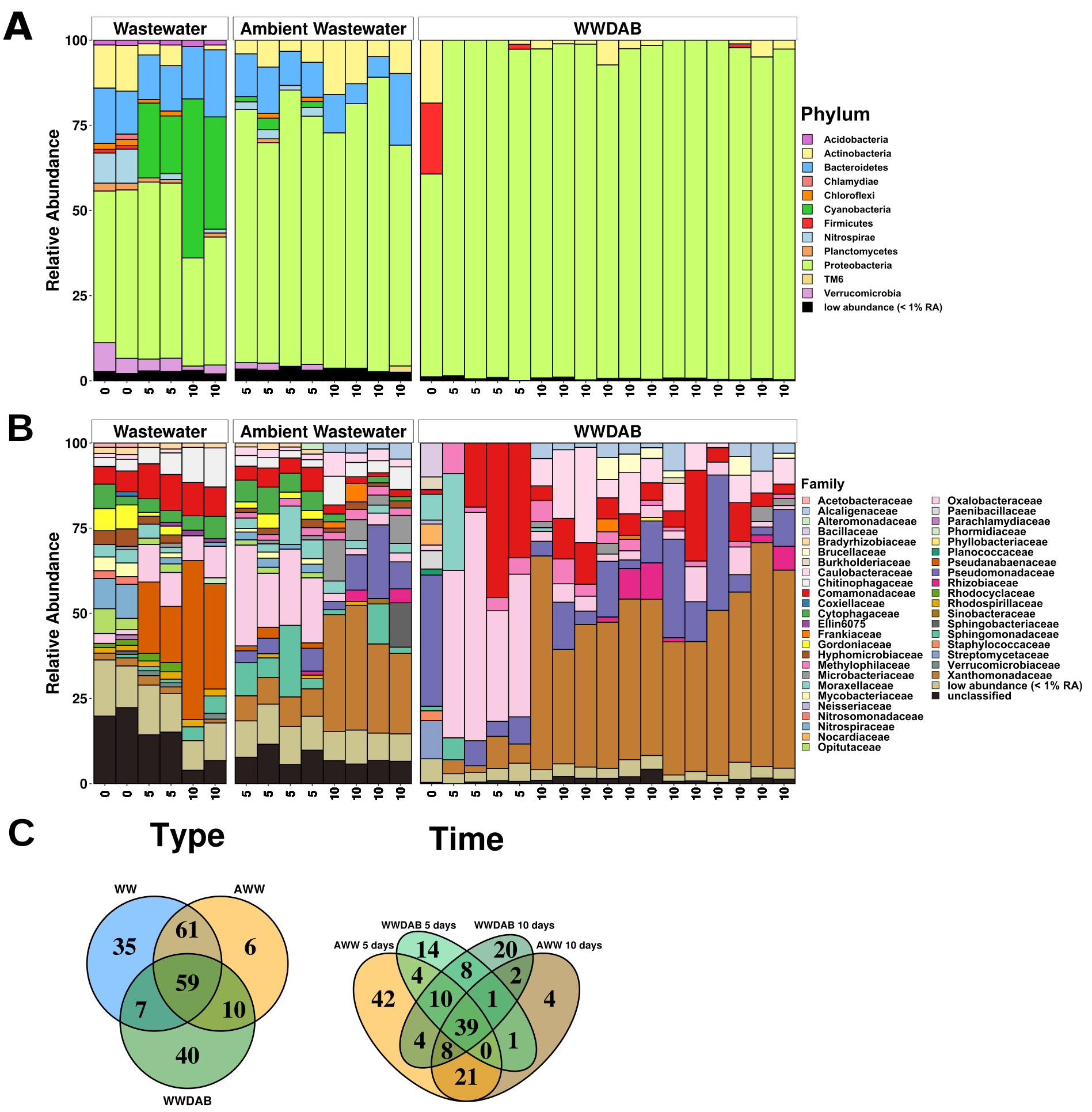

Supplement: S6 Fig — (A) Phylum, and (B) family level composition of Princeton Meadows year 1 bacterial communities. (C) Number of bacterial genera specific to and shared between bacterial communities and time points. (TIF) [file pone.0228560.s006.tif]

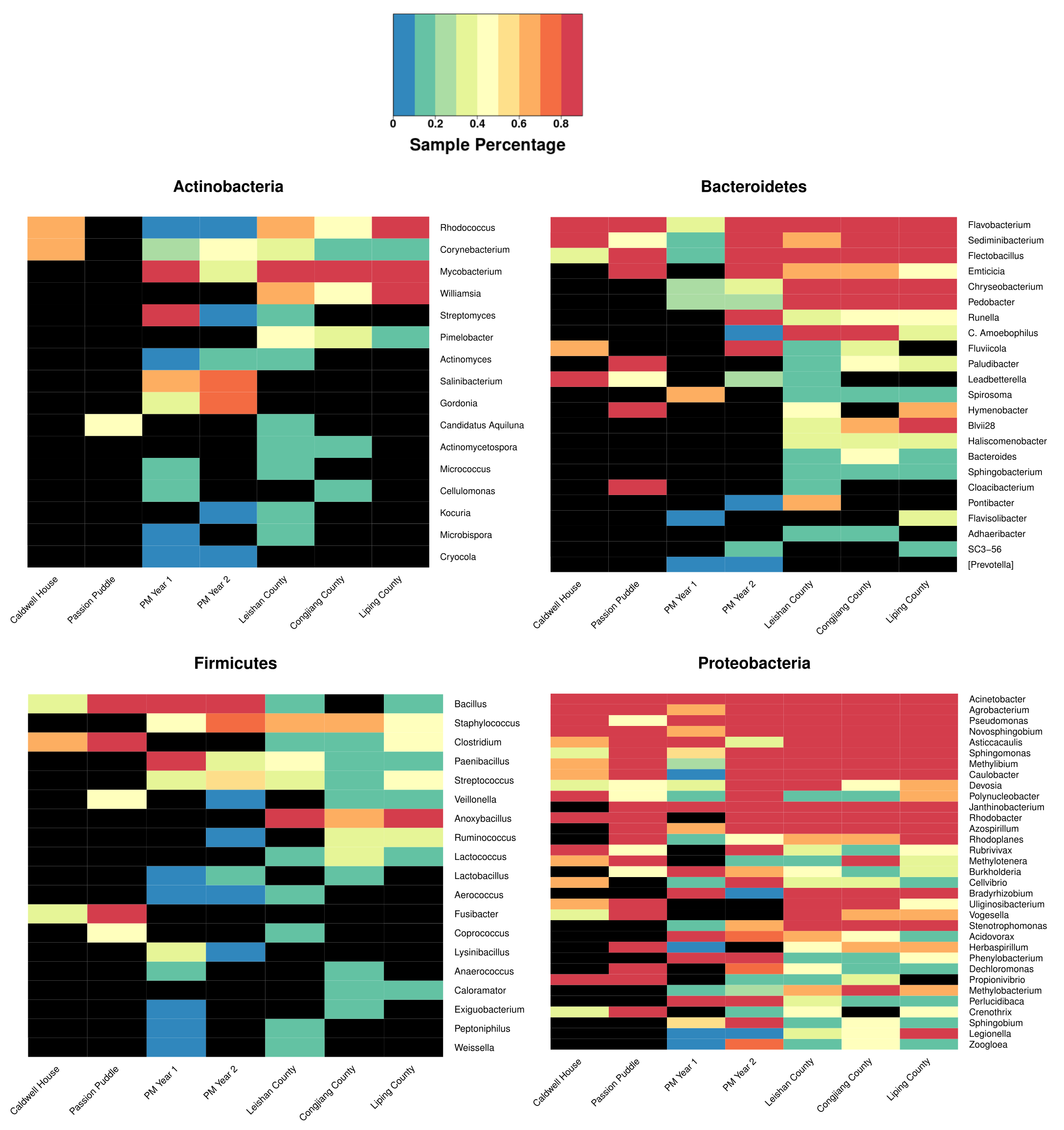

Supplement: S7 Fig — DAB communities from different studies were analyzed for the presence of bacterial taxa from the phyla Actinobacteria, Bacteroidetes, Firmicutes, and Proteobacteria. The percentage of samples each bacterial taxa was observed in for each location was calculated. The color black illustrated in heatmaps means bacterial taxa was not observed in any samples for that location. Actinobacteria, Bacteroidetes, and Firmicutes genera that were observed in more than 1 study are displayed while Proteobacteria taxa in more than 4 studies are displayed. (TIF) [file pone.0228560.s007.tif]
