# Supplementary material for: Duckweed hosts a taxonomically similar bacterial assemblage as the terrestrial leaf microbiome
Source: PLoS One. 2020 Feb 6;15(2):e0228560. doi: 10.1371/journal.pone.0228560 (PMC7004381; doi:10.1371/journal.pone.0228560)
Supplement: Attachment — Submitted filename: PONE-D-19-25118 with markup.pdf [file pone.0228560.s020.pdf]

PLOS ONE

Duckweed hosts a taxonomically similar bacterial assemblage as the terrestrial leaf microbiome

--Manuscript Draft--

**Manuscript Number:**	PONE-D-19-25118
**Article Type:**	Research Article
**Full Title:**	Duckweed hosts a taxonomically similar bacterial assemblage as the terrestrial leaf microbiome
**Short Title:**	Duckweed Bacterial Microbiome
**Corresponding Author:**	Eric Lam, Ph.D.<br><br>UNITED STATES
**Keywords:**	
**Abstract:**	Plant microbiota provide many benefits to the plant host including growth promotion and disease protection. While many plant microbial community profiling studies have been conducted on terrestrial plants much less is known about the aquatic plant microbiome. Duckweeds are small aquatic plants with many characteristics, including small size, reduced plant architecture, and aquatic habitat, that suggest their use as a facile model system for plant microbiome studies. We present here a comprehensive analysis of the duckweed bacterial microbiome from different environments. First, a culture-independent survey of the duckweed bacterial microbiome from different locations in New Jersey revealed similar phylogenetic profiles with minor qualitative variations. However, major differences in relative abundance of community members were observed between sites. These studies also showed that Proteobacteria is a dominant phylum in the duckweed bacterial microbiome. To observe the assembly dynamics of the duckweed bacterial community, we inoculated quasi-gnotobiotic duckweed with wastewater effluent from a municipal wastewater treatment plant. Our results revealed that duckweed strongly shapes its assembled bacterial microbiome and forms distinct associations with bacterial community members from the initial inoculum. Additionally, these inoculation studies showed the bacterial communities of different duckweed species contain a highly similar phylogenetic structure. Analysis of the duckweed bacterial community across several different studies identified a set of “core” bacterial genera consistently present on duckweed irrespective of the locale and context. Taxonomic comparison of the duckweed bacterial community to that in rice and Arabidopsis revealed a similar taxonomic structure between duckweed and the terrestrial leaf microbiome. Our results suggest that duckweeds utilize similar bacterial community structuring principles as those found in terrestrial plants. The information presented in this study will help inform the design of future experimental studies aimed at understanding plant microbial community establishment with the goal of leveraging plant-microbe interactions for improved growth performance.
**Order of Authors:**	Kenneth Acosta
	Jenny Xu
	Sarah Gilbert
	Thomas Brinkman
	Elizabeth Denison
	Sarah Lebeis
	Eric Lam
**Additional Information:**	
**Question**	**Response**
**Financial Disclosure**	Duckweed research in EL laboratory was supported in part by a grant from the Department of Energy (DE-SC0018244) and a Hatch project (#12116) from the New

Enter a financial disclosure statement that describes the sources of funding for the work included in this submission. Review the submission guidelines for detailed requirements. View published research articles from *PLOS ONE* for specific examples.<br><br>This statement is required for submission and **will appear in the published article** if the submission is accepted. Please make sure it is accurate.<br><br>**Unfunded studies**<br>Enter: *The author(s) received no specific funding for this work.*<br><br>**Funded studies**<br>Enter a statement with the following details:<br>• Initials of the authors who received each award<br>• Grant numbers awarded to each author<br>• The full name of each funder<br>• URL of each funder website<br>• Did the sponsors or funders play any role in the study design, data collection and analysis, decision to publish, or preparation of the manuscript?<br>• **NO** - Include this sentence at the end of your statement: *The funders had no role in study design, data collection and analysis, decision to publish, or preparation of the manuscript.*<br>• **YES** - Specify the role(s) played.<br><br>* typeset	Jersey Agricultural Experiment Station at Rutgers University. SL acknowledges start-up funds provided to her from the University of Tennessee, Knoxville. The funders had no role in study design, data collection and analysis, decision to publish, or preparation of the manuscript.
**Competing Interests**<br><br>Use the instructions below to enter a competing interest statement for this submission. On behalf of all authors, disclose any competing interests that could be perceived to bias this work—acknowledging all financial support and any other relevant financial or non-financial competing interests.<br><br>This statement **will appear in the published article** if the submission is accepted. Please make sure it is accurate. View published research articles from *PLOS ONE* for specific examples.	The authors have declared that no competing interests exist.

Powered by Editorial Manager® and ProduXion Manager® from Aries Systems Corporation

**NO authors have competing interests**<br><br>Enter: *The authors have declared that no competing interests exist.*<br><br>**Authors with competing interests**<br><br>Enter competing interest details beginning with this statement:<br><br>*I have read the journal's policy and the authors of this manuscript have the following competing interests: [insert competing interests here]*<br><br>* typeset	
**Ethics Statement**<br><br>Enter an ethics statement for this submission. This statement is required if the study involved:<br><br>• Human participants<br>• Human specimens or tissue<br>• Vertebrate animals or cephalopods<br>• Vertebrate embryos or tissues<br>• Field research<br><br>Write "N/A" if the submission does not require an ethics statement.<br><br>General guidance is provided below. Consult the submission guidelines for detailed instructions. **Make sure that all information entered here is included in the Methods section of the manuscript.**	N/A

**Format for specific study types**<br><br>**Human Subject Research (involving human participants and/or tissue)**<br>• Give the name of the institutional review board or ethics committee that approved the study<br>• Include the approval number and/or a statement indicating approval of this research<br>• Indicate the form of consent obtained (written/oral) or the reason that consent was not obtained (e.g. the data were analyzed anonymously)<br><br>**Animal Research (involving vertebrate animals, embryos or tissues)**<br>• Provide the name of the Institutional Animal Care and Use Committee (IACUC) or other relevant ethics board that reviewed the study protocol, and indicate whether they approved this research or granted a formal waiver of ethical approval<br>• Include an approval number if one was obtained<br>• If the study involved *non-human primates*, add *additional details* about animal welfare and steps taken to ameliorate suffering<br>• If anesthesia, euthanasia, or any kind of animal sacrifice is part of the study, include briefly which substances and/or methods were applied<br><br>**Field Research**<br><br>Include the following details if this study involves the collection of plant, animal, or other materials from a natural setting:<br>• Field permit number<br>• Name of the institution or relevant body that granted permission	
**Data Availability**<br><br>Authors are required to make all data underlying the findings described fully available, without restriction, and from the time of publication. PLOS allows rare exceptions to address legal and ethical concerns. See the PLOS Data Policy and FAQ for detailed information.	Yes - all data are fully available without restriction

A Data Availability Statement describing where the data can be found is required at submission. Your answers to this question constitute the Data Availability Statement and **will be published in the article**, if accepted.<br><br>**Important:** Stating 'data available on request from the author' is not sufficient. If your data are only available upon request, select 'No' for the first question and explain your exceptional situation in the text box.<br><br>Do the authors confirm that all data underlying the findings described in their manuscript are fully available without restriction?	
**Describe where the data may be found in full sentences. If you are copying our sample text, replace any instances of XXX with the appropriate details.**<br><br>• If the data are **held or will be held in a public repository**, include URLs, accession numbers or DOIs. If this information will only be available after acceptance, indicate this by ticking the box below. For example: *All XXX files are available from the XXX database (accession number(s) XXX, XXX.).*<br>• If the data are all contained **within the manuscript and/or Supporting Information files**, enter the following: *All relevant data are within the manuscript and its Supporting Information files.*<br>• If neither of these applies but you are able to provide **details of access elsewhere**, with or without limitations, please do so. For example:<br><br>*Data cannot be shared publicly because of [XXX]. Data are available from the XXX Institutional Data Access / Ethics Committee (contact via XXX) for researchers who meet the criteria for access to confidential data.*<br><br>*The data underlying the results presented in the study are available from (include the name of the third party*	Duckweed barcode sequences and 16rDNA amplicon libraries are available under the NCBI Bioproject ID PRJNA561628. All QIIME 2 and R code is available at https://github.com/kenscripts/duckweed_microbiome.

Powered by Editorial Manager® and ProduXion Manager® from Aries Systems Corporation

and contact information or URL).<br>• This text is appropriate if the data are owned by a third party and authors do not have permission to share the data.<br><br>* typeset	
Additional data availability information:	Tick here if the URLs/accession numbers/DOIs will be available only after acceptance of the manuscript for publication so that we can ensure their inclusion before publication.

Powered by Editorial Manager® and ProduXion Manager® from Aries Systems Corporation

Manuscript
Click here to access/download;Manuscript;2019-PLoS_One-Complete_Draft.submitted.docx

Duckweed hosts a taxonomically similar bacterial assemblage as the terrestrial leaf microbiome

Kenneth Acosta[1], Jenny Xu[1], Sarah Gilbert[1], Thomas Brinkman[1], Elizabeth Denison[2],Sarah Lebeis[2], Eric Lam[1*]

[1] Department of Plant Biology, Rutgers the State University of New Jersey, New Brunswick, New Jersey, United States of America

[2] Department of Microbiology, University of Tennessee, Knoxville, Tennessee, United States of America

* Corresponding author

E-mail: ericl89@hotmail.com (EL)

Abstract

Plant microbiota provide many benefits to the plant host including growth promotion and disease protection. While many plant microbial community profiling studies have been conducted on terrestrial plants much less is known about the aquatic plant microbiome. Duckweeds are small aquatic plants with many characteristics, including small size, reduced plant architecture, and aquatic habitat, that suggest their use as a facile model system for plant microbiome studies. We present here a comprehensive analysis of the duckweed bacterial microbiome from different environments. First, a culture-independent survey of the duckweed bacterial microbiome from different locations in New Jersey revealed similar phylogenetic profiles with minor qualitative variations. However, major differences in relative abundance of community members were observed between sites. These studies also showed that Proteobacteria is a dominant phylum in the duckweed bacterial microbiome. To observe the assembly dynamics of the duckweed bacterial community, we inoculated quasi-gnotobiotic duckweed with wastewater effluent from a municipal wastewater treatment plant. Our results revealed that duckweed strongly shapes its assembled bacterial microbiome and forms distinct associations with bacterial community members from the initial inoculum. Additionally, these inoculation studies showed the bacterial communities of different duckweed species contain a highly similar phylogenetic structure. Analysis of the duckweed bacterial community across several different studies identified a set of “core” bacterial genera consistently present on duckweed irrespective of the locale and context. Taxonomic comparison of the duckweed bacterial community to that in rice and Arabidopsis revealed a similar taxonomic structure between duckweed and the terrestrial leaf microbiome. Our results suggest that duckweeds utilize similar bacterial community structuring principles as those found in terrestrial plants. The information presented in this study will help inform the design of future experimental studies

aimed at understanding plant microbial community establishment with the goal of leveraging plant-microbe interactions for improved growth performance.

Introduction

Terrestrial plants harbor a multitude of microorganisms that can confer fitness advantages either through plant growth promotion or disease protection [1]. A longstanding strategy used by researchers has been to isolate and apply beneficial bacterial members with plant growth promoting ability (PGPB) to sustainably improve crop yield. However, efforts to observe plant-growth promoting behavior by PGPB in the field have seen limited success as a result of many factors including the inability of a single PGPB to form stable associations with plant hosts over time [2]. A greater understanding of the interactions occurring between plant hosts and their myriad associated microorganisms, as well as between the microbes themselves, will be necessary for improving PGPB field efficacy [3][4].

To address these issues a number of culture-independent studies using next-generation sequencing technologies have been conducted on terrestrial plants to characterize the plant microbiome [5]. These analyses show a consistent assemblage of bacteria from the phyla Actinobacteria, Bacteroidetes, Firmicutes, and Proteobacteria on both roots and leaves of terrestrial plants [6]. Moreover, a number of factors can affect the composition of the terrestrial plant-associated microbial community including plant compartment and soil inoculum as strong determinants as well as plant genotype, developmental stage, and cultivation practice as relatively minor determinants [7][8][9]. Reductionist approaches using gnotobiotic plants, culture collections of plant microbiota, and sterile soil matrix have begun to

resolve the complexity of interactions occurring in the plant microbiome [10][11]. While much has been unveiled about the terrestrial plant microbiome there is a lack of information on aquatic plant microbiomes.

Duckweeds are aquatic plants that belong to the family Lemnaceae and are composed of 5 genera and 37 species [12]. Duckweed has been used in many ecotoxicological and phytoremediation studies and there is growing interest in duckweed as biofuel, animal, and human feedstock [13][14]. The addition of duckweed genomic resources and molecular tools in the past few years has positioned duckweed as a model system for several aspects of plant biology [15-18]. Duckweed possesses several desirable characteristics that facilitate its use as a model system to study plant microbial communities. Duckweed has a simple plant architecture consisting of mainly leaf-like structures termed fronds while roots are only found in some species as simple roots with no lateral branching or root hairs. Duckweed is able to maintain this simple architecture throughout its life cycle since it mainly propagates clonally in the laboratory for the most part. Lastly, duckweed is only a few millimeters in size and one of the fastest growing plant species which enable economy of space and time for their study [19]. These traits can be leveraged to facilitate high-throughput microbiome studies as they were previously exploited to develop screens for microbial pathogenesis [20-21]. More importantly, its aquatic habitat is relatively homogenous compared to soil and allows for straightforward sampling in addition to accurate and robust measurements. As an angiosperm, duckweed may provide additional information on the evolution of plant microbiomes since the monocot ancestors for this plant family transitioned from terrestrial environments back to an aquatic lifestyle about 100 Mya [22].

Here, we present a comprehensive analysis of the duckweed bacterial microbiome. Results using 16S rRNA gene community profiling indicated the selection of particular duckweed-bacterial associations from the environment. These associations were constituted mostly by Proteobacteria. Duckweed bacterial communities sampled from different duckweed species showed similar phylogenetic signatures while quantitative differences were found between communities sampled from different locations. By combining results from several different studies, a set of “core” bacterial families and genera consistently associated with duckweed were identified. Comparison of the duckweed tissue bacterial community to that of rice and Arabidopsis leaf tissues revealed a taxonomically similar bacterial microbiome. The data presented in this work should help inform the application of bacterial microbiota for improving performance of duckweed-based applications in addition to facilitate the development of experimental approaches to understand plant microbial community establishment.

Methods

Survey of the Duckweed Bacterial Community

Sample Collection

To survey the duckweed bacterial microbiome both duckweed and ambient water samples were collected from two ponds located in New Brunswick, NJ (Passion Puddle) and Great Meadows, NJ (Caldwell House) (S1 Protocol, Fig 1).

Ambient water samples were passed through sterilized Miracloth to remove solids. Ambient water was then filtered through a 0.2 um 150 mL Nalgene rapid filter unit (ThermoScientific, Catalog No. 125-0020) to capture the microbial community. Filter

membranes were excised, placed in 5 mL centrifuge tubes, flash frozen in liquid nitrogen, and stored until processing.

Duckweed tissue was first separated from other solids. Duckweed samples were then washed with salt and detergent (1X PBS, 0.1% Triton X-100, 0.5 mM MgSO4, 1 mM CaCl2) and rinsed twice with water. Duckweed tissue was placed in 5 mL centrifuge tubes and flash frozen in liquid nitrogen. Samples were stored at -80°C until processing.

Duckweed Genotyping

Duckweed collected were genotyped using two barcodes: *atpF-atpH* (5'-ACTCGCACACACTCCCTTTCC-3' and 5'-GCTTTTATGGAAGCTTTAACAAT-3') and *psbK-psbI* (5'-TTAGCATTTGTTTGGCAAG-3' and 5'-AAAGTTTGAGAGTAAGCAT-3') as suggested in [23]. Different duckweed samples were surface sterilized using 10 % bleach, washed with 2 % sodium thiosulfate followed by water, and cultured on media containing 0.5X Schenk and Hildebrandt (SH) (Phytotechnology Laboratories, Catalog No. S816), 0.1 % sucrose, and 100 ug/mL cefotaxime. Duckweed are maintained and registered at the Rutgers Duckweed Stock Cooperative (RDSC).

Bacterial Community Inoculation Studies

Duckweed Tissue Propagation

L. minor 370-5576 (Lm5576) and *S. polyrhiza* 432-9509 (Sp9509) were obtained from the RDSC. Duckweed tissue was first propagated in 50 mL baby food jars containing sterile growth media (0.5X SH and 0.1 % sucrose at pH 7), then in 200 mL of growth media, and lastly in 400 mL of growth media for approximately two weeks at a time.

Sample Inoculation

For inoculation studies, wastewater from the United Water Princeton Meadows wastewater treatment facility in Plainsboro, NJ was collected after secondary clarification in the years 2015 (year 1) and 2016 (year 2).

For the Princeton Meadows year 1 study, 75 mL of Princeton Meadows wastewater collected in 2015 was inoculated with approximately 200 mg fresh weight Sp9509. Samples were collected at 0, 5, and 10 days at 26°C and a photo-peroid of 16 hr day and 8 hr night. Samples were harvested using 0.2 um Nalgene rapid-flow filter units. Miracloth was overlaid on filter unit to capture duckweed tissue. Duckweed tissue was transferred to 5 mL centrifuge tissue and washed with salt and detergent followed by sterile water two times. Tissue was then flash frozen with liquid nitrogen and stored at -80°C until processing. Wastewater and ambient wastewater samples were filtered through 0.2 um membrane to capture microbial community. Membranes were excised, placed in 5 mL centrifuge tubes, flash frozen in liquid nitrogen, and stored at -80°C until processing.

In the Princeton Meadows year 2 study 50 mL of Princeton Meadows wastewater collected in 2016 was inoculated with either Lm5576 or Sp9509. Princeton Meadows samples were harvested at 2 and 7 days. Duckweed tissue was either treated with water only or with salt and detergent followed by two water washes. Tissue and ambient wastewater samples were processed as mentioned above.

DNA Isolation and Library Preparation

Frozen duckweed tissue was homogenized for 15 minutes at 1500 RPM in the Geno/Grinder 2010 (SPEX SamplePrep) with approximately 20 sterile garnet beads (0.7mm, Qiagen). DNA was extracted using PowerSoil DNA Extraction Kit (MoBio) and stored at -80˚C.

For water samples, DNA was extracted from filters using the PowerWater DNA Extraction Kit (MoBiol). All DNA sample concentrations were quantified using the PicoGreen dsDNA kit (Invitrogen). To ensure that each sample contained amplifiable DNA, all samples had a 16S rRNA gene PCR performed using 515F and 806R primers as quality control before amplicon library construction.

Library preparation and sequencing was performed at the Joint Genome Institute as a part of the Community Science Program (Department of Energy, CSP project # DE-SC0018244). For library preparation, 25 µL reactions contained 11.4 µL PCR grade water, 1 µL BSA (10mg/ml), 10 µL 5 PRIME HotMaster Mix, 0.5µL of 16S rRNA gene primers (10 µM), 1µL of DNA (10ng/ µL), and 0.3 µL (100 µM) of a mixture of two Peptide Nucleic Acids (PNA) one for blocking plant mitochondrial sequences and one for blocking plant plastid sequences [24]. For each sample, reactions were run in triplicate. All samples were amplified with the primers 515F-Y (5'-GTGYCAGCMGCCGCGGTAA-3') and 926R (5'-CCGYCAATTYMTTTRAGTTT-3') [25]. The thermocycler settings were: 3 minutes at 93˚C, with 30 cycles of 94˚C for 45 seconds, 78˚C for 10 seconds (for PNA annealing), 50˚C for 60 seconds, 72˚C for 90 seconds and a 10 minute final extension at 72˚C. Triplicate samples were combined and run on a 1% agarose gel to confirm PCR success and cleaned using Agencourt AMpure XBeads (Beckman Coulture) in a 1:1 ratio of beads to product according to the protocol specified in Illumina's 16S Metagenomic Sequencing Library Preparation. Secondary PCR to index each sample with unique adapters was performed after cleaning. Reactions for Index PCR consisted of 25 µL of 10 µL 5 PRIMER HotMaster Mix, 11.4 µL of sterile PCR grade water, 0.5 µL of both Nextera XT Index Forward and Reverse primer (JGI primers), 5µL of cleaned DNA, and 0.3 µL (100 µM) of the 2 PNA mixture. The thermocycler settings were 94˚C for 3 minutes, with 8 cycles of 94˚C for 30 seconds, 78˚C for 10 seconds (for PNA annealing), 50˚C for 30 seconds, 72˚C for 30 seconds and 5 minute final extension at 72˚C. Indexing PCR success were visualized on 1% agarose

gels and samples were cleaned again according to the same magnetic bead based protocol from Illumina. After the final clean up, the DNA concentration of all samples were quantified using a PicoGreen Assay (Invitrogen) and pooled equally according to their DNA concentration. The library was then processed at the Joint Genome Institute. They were first run on a Bioanalyzer High Sensitivity Chip (Agilent Technologies) to quantify concentration and confirm amplicon size then sequenced using Version 2, 300 cycle (2 X 275) kit on the Illumina MiSeq platform.

Microbiome Bioinformatics

Creating Feature Tables and Classifiers Using QIIME2

For each experiment raw sequences were processed using QIIME 2 (q2) version 2018.6 [26]. Sequences were imported and demultiplexed using the SingleEndFastqManifestPhred33 Fastq manifest format. Quality control was performed and feature tables containing counts for the different amplicon sequence variants (ASVs) were produced using the q2-dada2 plugin [27].

ASV taxonomic classifiers were generated using the Greengenes 13_8 99 % OTUs reference database and the q2-feature-classifier plugin with classify-consenus-blast as the classification method [28-29]. The classify-consensus-blast method was chosen due to its ability to classify a large percentage of reads (S1 Fig.).

Feature tables were filtered for unclassified, mitochondria, chloroplast, and low frequency (> 1) ASVs (Fig S2).

Diversity, Taxonomic, and Differential Abundance Analysis

ASVs were aligned using the q2-alignment plugin [30] and phylogenies were constructed using the q2-phylogeny fasttree plugin [31]. The q2-diversity plugin was then used to rarefy each feature table and calculate the number of observed ASVs and Faiths' phylogenetic diversity [32]. Unweighted UniFrac, weighted UniFrac, and Bray-Curtis distance metrics were also generated. The generalized UniFrac distance metric was produced using the GUniFrac R package [33]. The q2-taxa barplot plugin was used to determine the taxonomic composition of feature tables at the Phylum and Family levels. Differential abundance testing was conducted using ALDEx2 [34].

Data Visualization, Statistics, Code, Protocols, and Data Availability

QIIME 2 artifacts were exported and data visualizations were created using R version 3.6.0. Statistics were performed using R where appropriate. All QIIME 2 and R code along with manifest, metadata, input, and output files are available at: https://github.com/kenscripts/duckweed_microbiome.

Protocols and workflows used in this study are available at: https://www.researchgate.net/profile/Kenneth_Acosta. Amplicon libraries and duckweed barcode sequences have been uploaded under the NCBI BioProject ID PRJNA561628.

Results

Survey of the Duckweed Bacterial Microbiome

To survey the composition of the duckweed bacterial microbiome, both duckweed and surrounding (ambient) water samples were collected during the summer months from two residential ponds in New Jersey (Fig. 1). A two-barcode strategy [23] was used to identify

duckweed species. Duckweed collected from the Caldwell House site were identified as *Wolffia brasiliensis* and duckweed from Passion Puddle were identified as *Lemna minor/japonica*. A much smaller quantity (< 1% in biomass) of *Lemna obscura* in addition to *Wolffia brasiliensis* was also found in the Caldwell House site. To examine the bacterial community of samples from the different sites, genomic DNA was extracted from our samples and the V4 region of the 16S rRNA gene was amplified and sequenced. A total of 13 samples were processed comprising of 3 duckweed samples and 4 ambient water samples from Caldwell House and 2 duckweed samples and 4 ambient water samples from Passion Puddle sites (S1A File). Due to the small size of duckweed entire plants were used for extraction and analysis. Reads were processed to remove low quality, chimeric, unclassified, chloroplast, and mitochondrial reads. Ambient water samples contained a median of 309148 reads and a range of 131305-350924 while duckweed samples comprised a median of 5684 reads with a range of 3664-12505. Amplicon sequence variants (ASVs) were tabulated for each sample using QIIME 2 resulting in 6002 bacterial ASVs with a total of 2,546,542 reads.

Figure 1. Sample collection sites. (A) Map of New Jersey, USA depicting sample collection sites. Duckweed and ambient water samples were collected from Caldwell House and Passion Puddle sites. Wastewater was collected from United Water Princeton Meadows treatment facility to use as an inoculum for inoculation studies. (B) Representative images of duckweed collected from Caldwell House and Passion Puddle sites.

To assess bacterial community diversity, cleaned data from samples were rarefied to a depth of 3664 reads (S2A Fig.). At this sampling depth, maximum diversity was captured in duckweed samples but some diversity was lost in water samples. Duckweed from both Caldwell House and Passion Puddle contained fewer amplicon sequence variants (ASVs) with an average of 129 ASVs compared to an average of 558 AVSs in ambient water (S3A Fig.).

Analysis of the phylogenetic diversity within samples using Faith's phylogenetic diversity index showed a lower phylogenetic index for the duckweed-associated bacterial community when compared to the ambient water bacterial community. Principal coordinate analysis (PCoA) of unweighted and generalized UniFrac distances were performed to compare the bacterial community diversity between duckweed and ambient water samples collected from two different locations (S3B Fig.). The unweighted UniFrac (UUF) does not consider taxa abundance and is sensitive to changes in rare taxa while the generalized UniFrac distance (GUF) is able to detect changes in abundance among moderately and highly abundant taxa [33]. Results from PCoA using both distance metrics revealed that the duckweed-associated bacterial community separates from the ambient water bacterial community along the first principal coordinate. The bacterial communities of ambient water samples showed a clear separation between locations along the second principal coordinate but did not cause as much division between the duckweed bacterial communities from the different locations. These data indicate that duckweed hosts a less diverse bacterial community than the surrounding water and is also phylogenetically similar between different locations, unlike that in the ambient water.

Taxonomic analyses on normalized data were performed at the phylum, family, and genus level to identify which bacteria associate with duckweed compared to ambient water (Fig. 2 and S2 File). Figure 2A illustrates that the phylum of Proteobacteria is a dominant constituent of the duckweed microbiome (82 % and 90% in Caldwell House and Passion Puddle sites respectively) compared to the ambient water (47 % in both Caldwell House and Passion Puddle sites). Some compositional differences at the family level were observed in the duckweed-associated community between locations as well as between replicates from each site (S2B File), however the Comamonadaceae family was the most abundant family in both Caldwell House and Passion Puddle duckweed (24 % and 25 % average relative abundance respectively). A total of 101 bacterial genera were found in the Caldwell House ambient water

but only 31 of these bacterial genera were found on duckweed while Passion Puddle ambient water contained 189 bacterial genera and only 41 were found on Passion Puddle duckweed (Fig. 2C). It is possible that those bacterial genera with a low relative abundance may not have been captured by sequencing while bacterial genera present in a low sample percentage could represent random associations. Additionally, those genera with a high sample percentage and not present in ambient water may have been acquired from another inoculation source such as the phyllosphere (S2E-F File). *Rhodobacter, Agrobacterium, Hydrogenophaga, Bacillus,* and *Novosphingobium* contained the top 5 mean relative abundances and were found in all Caldwell House duckweed samples while *Rhizobium, Dechloromonas, Sphingomonas, Agrobacterium*, and *Sulfurospirillum* were found in all Passion Puddle samples and held top mean relative abundances (S2C-D File). Caldwell House and Passion Puddle duckweed shared 28 bacterial genera while 10 bacterial genera were specific to Caldwell House duckweed and 22 bacterial genera were specific to Passion Puddle duckweed (Fig. 2C, S2G-H File). Of the 22 Passion Puddle-specific duckweed-associated bacterial genera 11 were found in the Passion Puddle ambient water (Fig. 2C). This included the genus *Rhizobium* which held the highest mean relative abundance among Passion Puddle duckweed-associated bacteria (S2H File). Unique methods have been developed for differential abundance testing in order to deal with the constraints imposed by compositional data such as that from 16S amplicon sequencing [35]. ALDEx2 was selected for differential abundance analysis because of its low false discovery rate and simplicity [36]. ALDEx2 transforms counts or abundances into a distribution of log 2 centered-log ratios (clr). Its output includes effect size and significance testing to identify features that are different between groups. ALDEx2 was used to evaluate which bacterial genera are enriched on duckweed compared to ambient water in both locations (S3 File). ALDEx2 determined 7 bacterial genera were significantly different in abundance between Caldwell House duckweed tissue and ambient water with 4 of these genera being enriched on duckweed (Fig. 3). Passion Puddle duckweed contained 7 genera that were significantly

different in abundance compared to ambient water and all were enriched on duckweed. Different bacterial genera were enriched on duckweed between locations. Together, these results show that only particular duckweed-bacteria associations are selected from the surrounding water and these associations are quantitatively and to some extent qualitatively different between locations.

Fig 2. Proteobacteria are the major constituent of the duckweed bacterial microbiome. Total bacterial (A) Phylum, (B) Family, and (C) genus-level taxonomic composition of duckweed and ambient water samples collected from Caldwell House and Passion Puddle sites. AW = ambient water; DK = duckweed; CH = Caldwell House; PP = Passion Puddle.

Fig 3. Different bacterial genera are enriched in duckweed between Caldwell House and Passion Puddle sites. ALDEx2 was performed to determine differentially enriched bacterial genera between duckweed and the ambient water in (A) Caldwell House and (B) Passion Puddle. The distribution of log2 centered-log ratios (CLR) for bacterial genera that were found to be significantly different between duckweed and ambient water samples are displayed using violin plots ("*" = adjusted Welch's t-test, p-value < 0.05). Effect sizes are displayed for each bacterial genus. Larger values signify a greater difference between sample types. Positive effect sizes represent a higher abundance in duckweed compared to ambient water while negative effect sizes represent a higher abundance in ambient water compared to duckweed.

Assembly Dynamics of the Duckweed Bacterial Community

The Princeton Meadows year 1 study was conducted to investigate the factors that drive the composition and establishment of a duckweed-associated bacterial community. In this study, repeatedly bleach-treated *S. polyrhiza* 9509 (Sp9509) was inoculated with Princeton

Meadows municipal wastewater effluent collected in 2015 (Fig. 1 and S1B Fie). To test for the effect of sample type on the bacterial community 3 different types of samples were collected: wastewater that was not co-cultured with Sp9509 (wastewater), wastewater co-cultured with Sp9509 (ambient wastewater) and the respective Sp9509 tissue. Samples were collected at 0, 5, and 10 days to investigate the effect of time on the association of microbes from the wastewater with the “cleaned” duckweed tissue. A total of 31 samples were collected with 6 wastewater samples (median of 240155 reads with range of 184943-30921), 8 ambient wastewater samples (median of 224388 reads with range of 139299-302196), and 17 duckweed samples (median of 158908 reads with range of 6262-206513) resulting in 5793 bacterial ASVs encompassing 5,835,517 reads. Despite taking measures to ensure duckweed sterility after our bleach treatments, such as 1) plating duckweed onto solid LB media and checking for microbial growth at 28°C for up to 3 days and 2) conducting PCR using 16S-23S IGS primers to detect bacteria DNA, amplicon sequencing of initial Sp9509 tissue nevertheless captured 151471 reads with 6580 reads belonging to 83 bacterial ASVs and from which (6262 reads were able to be classified at the phylum level (S4A File). However, the number of bacterial ASVs found in this initial Sp9509 tissue (designated as “quasi-gnotobiotic”) was significantly lower (Wilcoxon rank sum test, p-value < 0.05) when compared to tissues several days after inoculation with wastewater (S4 Fig). Of the 83 bacterial ASVs 35 were specific to the initial Sp9509 tissue and remained present on duckweed after growing on wastewater (S5A Fig.) and only 11 bacterial ASVs, encompassing 6 bacterial genera, contained higher than average abundance at 10 days after wastewater inoculation (S4B File and S5B Fig). The stability of these bacterial ASVs indicate a strong association with Sp9509 and may explain the difficulty encountered in sterilization of the initial Sp9509 tissue. Surprisingly, a total of 884 bacterial ASVs were found only in wastewater-inoculated Sp9509 and not in the initial wastewater inoculum or initial Sp9509 tissue (S5A Fig.). More than half of these bacterial ASVs (64 %, 566 ASVs) were found in only 1 wastewater-inoculated Sp9509 sample while 23 % (203 ASVs) were found in less than

25 % of wastewater-inoculated Sp9509 samples (S5C Fig.). These 769 bacterial ASVs contained a less than average abundance (median CLR < 0) (S4C File). The remaining 115 (13 %) of wastewater-inoculated Sp9509 bacterial ASVs were found in greater than 25 % of samples with 19 bacterial ASVs containing a higher than average abundance (median CLR > 0) (S4C File). In comparison, 103 out of 237 (44 %) of bacterial ASVs that were in both wastewater-inoculated Sp9509 and initial wastewater were found in greater than 25 % of wastewater-inoculated Sp9509 with 80 bacterial ASVs containing a higher than average abundance (median CLR > 0) (S5C Fig. and S4C File). These data show that many of the bacterial ASVs specific to wastewater-inoculated Sp9509 are sporadic and in low abundance and suggests that these bacterial ASVs may have been in too low of abundance in wastewater to be detected since sufficient sequencing depth of wastewater samples was performed to reach saturation of bacterial ASV detection (S2B Fig.). Additionally, while many wastewater-inoculated Sp9509 bacterial ASVs were not able to be classified at the genus level, bacterial ASVs found in greater than 25 % of samples were mostly from the same Proteobacteria families including Commamonadaceae, Oxalobacteraceae, and Xanthomonadaceae (S4C File). These data suggest a preferred association between bacterial ASVs from certain Proteobacteria families and duckweed.

Diversity analyses were performed on rarefied samples (112500 reads) to examine sample diversity and determine which factors are critical for the assembly of the duckweed bacterial community (S2B Fig). Calculation of the number of ASVs observed and of Faith's PD index within samples indicate that only a subset of the microbiota in the wastewater was assembled into quasi-gnotobiotic Sp9509 (Fig. 4A) similar to natural communities found in wild duckweed tissue and their environmental microbiota (S3 Fig). Between-sample diversity analyses, using both UUF and GUF distances, show sample type is the main factor driving community composition (Fig 4B). Time was shown to influence the abundance of different

bacterial members in the wastewater and ambient wastewater along the second coordinate using the GUF distance but not on the duckweed-associated community. Diversity analyses from this inoculation study reiterate findings from the natural community study and support the observation that only certain bacteria from the environment can stably associate with duckweed.

Fig 4. Distinct duckweed-bacteria associations are selected from wastewater bacterial community. (A) Calculation of the number of ASVs and Faith's PD index of wastewater, ambient wastewater, and Sp9509 samples from Princeton Meadows year 1 study (Wilcoxon rank sum test; p-value < 0.05 = "*", p-value < 0.01 = "**", p-value < 0.001 = "***", p-value $<$ 0.0001 = "****"). (B) Principle coordinate analyses using unweighted and generalized UniFrac distances.

Taxonomic analyses and differential abundance testing were performed to identify significant changes in composition caused by different factors. Similar to the natural bacterial community found in wild duckweed tissues, taxonomic analysis showed Proteobacteria prevalently assemble onto duckweed (95 % mean relative abundance) (S6A Fig.). Differences in bacterial community composition were observed at the family level not only between sample type but also between different time points (S6B Fig.). At the genus level, some bacterial genera were not shared between sample type and time points (S6C Fig.). Further analysis showed these bacterial genera were found in a small number of samples and most likely represent either bacterial genera that were not captured by sequencing or random associations (S5C-E File). Differential abundance testing using ALDEx2 found a total of 55 bacterial genera to be differentially enriched between the different sample types (S6A File, adjusted Kruskal-Wallis, $p < 0.05$ and general linearized model, $p < 0.05$). Pairwise comparison revealed 13 bacterial genera differed significantly in abundance between ambient wastewater and wastewater with 7 depleted and 6 enriched bacterial genera in ambient wastewater compared to

wastewater (S6B File, adjusted Welch's t-test p < 0.05, absolute effect size greater than 1.25). Sp9509 tissue showed 12 enriched and 33 depleted bacterial genera when compared to wastewater (S6C File, adjusted Welch's t-test p < 0.05, absolute effect size greater than 1.25). Comparison between Sp9509 tissue and ambient wastewater revealed 6 bacterial genera were enriched and 23 bacterial genera were depleted on Sp9509 compared to ambient wastewater (S6D File, adjusted Welch's t-test p < 0.05, absolute effect size greater than 1.25). These results indicate that several bacterial genera decrease in abundance in Sp9509 and indicate that only distinct bacterial genera form associations with Sp9509. Interestingly, it was found that bacteria enriched in Sp9509 compared to wastewater were also enriched in ambient wastewater (Fig 5). A few bacterial genera were enriched on Sp9509 that were not enriched in ambient wastewater but many of these were also present on initial Sp9509 tissue and are most likely enriched as a result of initial stable ASVs (S5 Fig. and S4 File). Pairwise comparison between 5 day and 10 day samples in Sp9509 samples found that the bacterial genus *Rhodanobacter* was enriched while *Janthinobacterium* was depleted at 10 days compared to 5 days (adjusted Welch's t-test, p-value < 0.05, absolute effect size > 1.25) (S7B File).

Fig 5. Bacterial genera enrichment on duckweed correlates with their enrichment in ambient water. ALDEx2 determined 12 significantly enriched bacterial genera in Sp9509 compared to wastewater (adjusted Welch's t-test, p-value < 0.05, absolute effect size greater than 1.25). The ALDEx2 distribution for each of these bacterial genera was then visualized for each sample type. ALDEx2 adjusted Welch's t-test p-values are displayed (p-value < 0.05 = "*", p-value < 0.01 = "**", p-value < 0.001 = "***", p-value < 0.0001 = "****"). CLR = log 2 centered log ratio

Using Princeton Meadows wastewater collected in year 2 study the impact of duckweed species on bacterial community assembly and composition was studied (S1C File). Princeton

Meadows wastewater collected in the summer of 2016 was inoculated with either *L. minor* 5576 (Lm5576) or *S. polyrhiza* 9509 (Sp9509). Ambient wastewater and duckweed tissue samples were collected at 2 and 7 days. Plant samples are commonly treated to compartmentalize the plant microbiome. To observe the effect of sample pre-treatment, duckweed samples were treated with either water to wash off any carry-over from the ambient wastewater and loosely attached bacteria or treated with salt and detergent solution (SD). Those bacteria remaining after SD treatment are assumed to represent strongly attached epiphytes and/or endophytes. A total of 3 ambient wastewater samples were collected with a median of 369247 reads (range of 333444-448324) and 19 duckweed samples with a median of 244098 reads (range of 2689-407149). This resulted in 5,312,976 reads composed of 4493 ASVs. ~~While a~~nalysis of amplicon data showed initial duckweed samples contained a median of 3155 bacterial reads encompassing 307 bacterial ASVs (S8A File)~~, t~~his quantity was significantly lower when compared to wastewater-inoculated duckweed samples when referenced to the background plastid and mitochondria derived reads (S4 Fig). Out of these 307 bacterial ASVs 85 were found in wastewater-inoculated duckweed (S7A Fig.). Analysis across the different time points found only 40 bacterial ASVs were stable; defined as those ASVs which contained a higher than average abundance at both 0 and 7 days (S7B Fig. and S8B File). Most of these stable bacterial ASVs came from Proteobacteria with members from the families Comamonadaceae, Oxalobacteraceae, and Pseudomonadaceae.

A generalized linear model was constructed encompassing the different factors in order to observe their impact on community composition at the genus level (S9A File). The model found a significant difference for abundance for 2 bacterial genera between the duckweed species we compared, 5 bacterial genera between the different time points, and no bacterial genera changed in abundance between plant chemical treatments (glm, p-value < 0.05). In contrast to the generalized linear model, pairwise comparison between Lm5576 and Sp9509

samples revealed only the *Xanthobacter* bacterial genus to be significantly different in abundance in these two hosts upon co-cultivation with the same wastewater under out conditions (S9B File, adjusted Welch's t-test, p-value < 0.05, effect size greater than 1.25). Inspection of amplicon reads from our initial duckweed samples did not reveal *Xanthobacter* in starting Lm5576 and Sp9509 tissues and suggests *Xanthobacter* may have been selected for by Lm5576 from the wastewater community (S8A File). Taxonomic analysis showed Lm5576 and Sp9509 shared 98 genera in their tightly associated microbiota (S8C Fig.). Many of the bacterial genera that were found to be specific to either Lm5576 or Sp9509 displayed a low mean relative abundance and sample percentage (S10A-B File). PCoA using both phylogenetic and Bray-Curtis distances did not reveal any clear separation between host duckweed species (Fig. 6A and S9 Fig.). Examination of bacterial genus abundance resulted in similar values for each genus between the two duckweed species studied (Fig 6B). Together, these data indicate that the duckweed species *S. polyrhiza* and *L. minor* likely host similar bacterial communities.

Fig 6. Different duckweed species host phylogenetically similar bacterial communities. (A) PCoA using unweighted and generalized UniFrac distances of samples from Princeton Meadows year 2 inoculation study. (B) Median log 2 centered-log ratios (CLR) of bacterial genera abundance from Lm5576 and Sp9509 duckweed samples were calculated using ALDEx2 output. Pairwise spearman rank correlation coefficient and p-value are displayed.

While the generalized linear model proposed 5 bacterial genera differed in abundance between time points pairwise comparison between the different time points determined that 15 bacterial genera changed in abundance between 2 and 7 days (S9C File). Interestingly, *Janthinobacterium* ~~was found to~~ decrease in abundance at later time points similar to the finding from the Princeton Meadows year 1 study. Taxonomic analysis showed that bacterial genera that were only found on duckweed at 2 days or 7 days contained a low sample percentage (S8C

Fig., S10C-D File). Diversity analysis showed time as a main factor affecting community composition out of the factors tested (Fig. 6A). Washing duckweed tissue with either water or salt and detergent did not cause any observable qualitative or quantitative differences in community composition (Fig. 6A) and pairwise comparison did not detect any differences (S9D File, adjusted Welch's t-test, p-value < 0.05). Taxonomic analysis showed bacterial genera specific to either water-treated or SD-treated duckweed were found in a low sample percentage and thus may represent noise in our assays (S8C Fig and S10E-F File).

Comparative Analysis of Bacterial Communities

Previous studies have suggested the presence of "core" plant microbiota [7-8], a group that represents microbes that are consistently found in the plant microbiome. Here, we conducted a cross-study comparison of duckweed-associated bacteria consortium in order to reveal any core members in the duckweed microbiome. Core bacterial microbiota were defined as those bacterial members ~~who~~ that were present in samples from all the compiled studies. We included Data from the Caldwell House, Passion Puddle, Princeton Meadows year 1, and Princeton Meadows year 2 studies along with data from an ecological study comparing rice tissues and duckweed microbiome in Chinese rice paddies ~~were included~~ **(Huang et al, unpublished)**. ~~Since t~~ The Lemnaceae ~~family has~~ diverged from the ~~major~~ monocot lineage~~, which includes rice, of angiosperms~~ more than 100 Mya when it returned to a completely aquatic habitat [22]~~,~~. ~~w~~ We would expect that it may have evolved novel associations with microbial partners in this type of environment while ~~retaining other conserved ones~~ conserving others. Results using this approach revealed a set of 13 "core" bacterial genera that are consistently present on duckweed tissues irrespective of their habit of origin (Fig. 7). Some genera from Bacteroidetes were found but Proteobacteria mainly constituted this core microbiome. These core bacteria were frequently possessed an above average abundance on duckweed but this abundance varied between studies (Kruskal-Wallis,

[So the core genera were not necessarily the most abundant? If so, this might be worth stating more explicitly.]

p-value < 0.05) (S10 Fig.). It should be noted that cross study comparison also revealed many different bacterial genera between studies (S1 Table).

Fig 7. A set of core bacteria are consistently found associated with duckweed. Duckweed samples from different studies were analyzed for the presence of bacteria from the phyl~~um~~a Actinobacteria, Bacteroidetes, Firmicutes, and Proteobacteria. The percentage of samples each bacterial genus was observed in for each location was calculated. Core bacteria represent bacterial genera that were observed in all 7 studies. ~~The color b~~Black ~~illustrated in heatmaps~~ denotes ~~means~~ bacterial gen~~us was~~era not observed in any samples for that location. Actinobacteria, Bacteroidetes, and Firmicutes genera that were observed in more two or ~~than 1~~ stud~~y~~ies are displayed while Proteobacteria genera in more three or ~~than 2~~ studies are displayed.

~~To observe differences between aquatic and terrestrial plant bacterial microbiomes a~~ We taxonomically compar~~ison was performed between~~ed duckweed-, rice-, and *Arabidopsis*-associated bacterial communities**.** Data from the studies mentioned above were used for duckweed-associated bacterial communities resulting in a total of 59 duckweed samples (S11 File). For the rice comparison, [9] ~~the Edwards et al study [9] was used where~~ root rhizosphere, rhizoplane, and endosphere fractions were combined resulting in a total of 137 root samples. Another rice study was compared containing an additional 14 root and 18 aerial tissue samples (Huang et al, submitted). Two studies were used for *Arabidopsis* where rhizosphere, epiphytic, and endophytic root and leaf fractions were combined resulting in a total of 48 root and 22 leaf samples [37-38]. Diversity analysis ~~showed the~~ separat~~ion of~~ed the respective plant-associated bacterial communities into three groups encompassing: 1) the leaf-associated bacterial community of all three hosts, 2) the rice root-associated bacterial community, and 3) the *Arabidopsis*-associated bacterial community (Fig 8A), and suggest a conserved structuring effect of leaf tissue on the plant-associated bacterial microbiome. Actinobacteria, Bacteroidetes,

Firmicutes, and Proteobacteria are the predominant phyla in the plant-associated bacterial microbiome. Analysis of the quantity of bacterial genera from these phyla found on the respective plant samples showed a significantly lower quantity of taxa from Actinobacteria on duckweed compared to terrestrial plant root samples (Fig 8B). While rice leaf tissue also contained a lower quantity of Actinobacteria, Arabidopsis leaf tissue maintained similar levels when compared to terrestrial plant root samples. Analysis of Arabidopsis and rice core microbiomes demonstrated more diverse core microbiota than duckweed. As defined above, the rice core microbiome was composed of 7 bacterial phyla and 54 bacterial genera (S11 Fig) while the Arabidopsis core microbiome consisted of 43 bacterial genera from Actinobacteria, Firmicutes, and Proteobacteria (S12 Fig). Interestingly, only 1 Actinobacteria genus constituted the rice core microbiome compared to 8 Actinobacteria genera in the Arabidopsis core microbiome.

Fig 8. Duckweed bacterial microbiome resembles the terrestrial leaf microbiome. The taxonomic composition at the genus level of duckweed, rice, and Arabidopsis bacterial communities were compared. Duckweed bacterial community data from 7 different studies were used resulting in 59 plant samples. Rice bacterial community data from [9] and our recent field study from rice roots and aerial tissues above rice paddies (Huang et al, submitted) were used resulting in a total of 151 root samples (rhizosphere, rhizoplane, and endosphere fractions were combined) and 18 leaf samples. An Arabidopsis study [38] was used for comparison where epiphytic and endophytic leaf fractions were combined for a total of 22 plant samples. Another Arabidopsis study was used [37] where rhizosphere and endosphere fractions from root tissues were combined resulting in a total of 48 root samples. For comparison samples were rarefied to 1000 reads. (A) PCoA analysis of bacterial genera abundance using the Bray-Curtis distance for bacterial community samples collected from duckweed, rice, and Arabidopsis. (B) The number of bacterial genera from the four predominant plant-associated bacterial phyla was calculated

for the different plant tissues. Each data point represents a plant sample and the number of bacterial genera found in that sample for a particular phylum. Multiple pairwise comparison testing was performed using Dunn's test with Benjamini-Hochberg adjustment and the resultant compact letters displayed on the top of the chart above each dataset.

Discussion

Factors Driving Duckweed-Associated Bacterial Community Assembly

~~The studies presented in this investigation sought to~~ We compared the impact of several different variables on the structure of the duckweed-associated bacterial community. ~~One apparent observation is that d~~Duckweed can strongly shape its bacterial community. Comparison of bacterial community phylogeny across different samples encompassing several different variables show duckweed ~~exerted the strongest influence on~~ supported a distinct community composition and ~~comprised a different~~ phylogenetic structure than ~~what is found in~~ the surrounding water (Fig 4, Fig 6, Fig S3, Fig S11). Taxonomic analysis between sample types showed only 31 bacterial genera were found on Caldwell House duckweed compared to 101 found in ambient water, 41 bacterial genera were found on Passion Puddle duckweed out of 189 in ~~the respective~~ ambient water, 69 bacterial genera were found on PM Year 1 duckweed from 136 in wastewater, and 162 bacterial genera associated with PM Year 2 duckweed out of 192 bacterial genera in ambient wastewater. Moreover, comparative analysis incorporating 7 different studies identified a set of core bacterial microbiota consistently found to associate with duckweed across a large range of samples from different locales and conditions. These findings suggest recruitment of the duckweed bacterial assemblage is not a random process and only distinct duckweed-bacterial associations are selected from the inoculum. This selection process has been unveiled

in many terrestrial plant species [7][9]**.** A landmark plant microbiome study conducted on rice clearly demonstrated this multi-step selection process where microbial diversity decreased from soil to rhizosphere to rhizoplane to endosphere [9]. Interestingly, a similar pattern was observed in our PM Year 1 study where bacteria diversity decreased and separated along wastewater, ambient wastewater, and duckweed respectively (Fig 4). Authors from the rice study additionally observed a majority of bacteria enriched in the rhizoplane/endosphere were also enriched in the rhizosphere of rice roots. In the PM Year 1 study presented here a similar recruitment dynamic was observed where bacteria enriched in duckweed from wastewater were also enriched in ambient wastewater compared to wastewater (Fig 5).

Soil site or origin has been found to be a major determinant of bacterial community composition serving as the initial inoculum for plant microbiome acquisition [7-9][39]. To observe the effect of inoculum origin on duckweed bacterial community structure we conducted a culture-independent survey of two natural sites (Fig 1). PCoA using the unweighted UniFrac distance showed a clear separation in the ambient water bacterial communities between locations but duckweed bacterial communities clustered together (Fig S3C). PCoA using the generalized UniFrac showed the same pattern in ambient water samples and a greater degree of separation between the duckweed bacterial communities (Fig S3D). These results suggested a similar phylogenetic profile between the duckweed bacterial communities collected from different sites. However, taxonomic analysis showed a few bacterial genera were present in Passion Puddle ambient water and duckweed that were not present in Caldwell House samples (Fig 2). Some of these bacterial genera contained a high mean relative abundance among Passion Puddle duckweed including the genus *Rhizobium* (File S2). Moreover, the total number of different bacterial genera between different duckweed studies was calculated and showed many duckweed-associated bacterial genera differed between studies (S1 Table). The Princeton Meadows studies, both year 1 and year 2, contained the largest number of different

bacterial genera when compared to other studies. One possible explanation for this is the large duckweed sample size from these two studies with wastewater in contrast to the smaller duckweed sample size in the other studies. This larger sample size may have resulted in a greater number of low abundan~~t~~ce bacterial genera to be captured that may have not been possible in the other studies. In summary, inoculum origin/location seems to contribute both quantitative and qualitative differences to bacterial community structure in both aquatic and terrestrial plants.

In the PM Year 2 study two different duckweed species, *L. minor* and *S. polyrhiza*, were inoculated with the same wastewater effluent. Beta diversity analyses of their associated bacterial communities did not show any clear separation in phylogenetic structure between the two species (Fig 6A). Examination of possible quantitative differences in bacterial community member abundance between the two species, using differential abundance analysis, did not detect major differences (File S9B). ~~Comparison of b~~Bacterial community member abundance between the two species was highly similar (Fig 6B, spearman rank coefficient = 0.79). This similarity extended down to ASVs since PCoA using the Bray-Curtis distance metric, which takes into account ASV abundance, did not show division between the two species (S9 Fig). Several terrestrial plant studies have investigated the effect of host species on community composition. ~~Comparison of~~ *Arabidopsis* ecotypes and species ~~to~~ and the sister species *C. hirsuta* which diverged 35 million years apart, showed ~~found~~ mostly quantitative differences in bacterial community composition [8]. ~~Investigation of the bacterial assemblage of r~~Rice ~~found that plant~~ genotype explained the least variance in bacterial community composition compared to the other factors tested [9]. Therefore, ~~data acquired in~~ this study and ~~respective~~ terrestrial plant microbiome studies show plant host species is a minor determinant for both aquatic and terrestrial plant bacterial community composition.

Both PM year 1 and PM year 2 inoculation studies unveiled sampling time to have an influence on bacterial community member abundance (Fig 4, Fig 6, Fig S6, File S9A). ~~Edwards et al. performed t~~Time-series experiments involving rice ~~and~~ similarly ~~observed~~ showed a strong effect of time on community composition. This temporal change in duckweed bacterial community composition may be attributed to either changes in the environment such as nutrient concentrations or dynamic host-microbe or microbe-microbe interactions.

Structure of Duckweed-Associated Bacterial Microbiome

Bacterial community profiles of duckweed collected from natural sites and inoculated with wastewater effluent revealed Proteobacteria as the prevailing phyl~~a~~um in the duckweed bacterial microbiome (Fig 2, Fig 7, Fig S5, Fig S10). However, between studies different bacterial genera from this phylum were enriched on duckweed compared to ambient water (Fig 3, Fig 5, Fig 6, Fig 7). While many ~~bacterial~~ genera from this phylum were only present in some of our studies, there were ~~9~~ nine core Proteobacteria genera that were associated in duckweed samples from all ~~7 different~~ seven duckweed studies (Fig 7). The abundance of ~~these~~ core bacterial genera also varied between ~~these~~ datasets (Fig S13). Recent studies using culture collections of plant-associated bacterial microbiota have identified critical features and roles for plant bacterial community members. Genomic analysis of bacterial microbiota genomes from *Arabidopsis* showed Proteobacteria contained the highest functional diversity compared to other plant-associated bacterial phyla [10]. Proteobacteria formed distinct functional clusters based on family taxonomy rather than by ecological niche. Further investigation identified carbohydrate and xenobiotic degradation as enriched gene categories in plant-associated microbiota. In line with proposed plant microbiome acquisition models these enriched gene categories may allow plant microbiota to establish a presence in the surrounding environment prior to their association with the respective plant host. *Pseudomonas* and *Acinetobacter* were two prominent

Proteobacteria genera found in the "core" duckweed bacterial microbiome (Fig 7). Recent investigations involving synthetic communities revealed *Pseudomonas* as one of the key bacterial genera involved in plant-microbiota mediated immunity against filamentous eukaryotic microbes [11][40]. Studies with duckweed inoculated with an *Acinetobacter* strain showed it was able to promote duckweed growth under sterile and non-sterile conditions, provide protection against indigenous microbes, and increase bioremediation capability through the degradation of phenol [41-42]. The omnipresence of Proteobacteria across duckweed and terrestrial bacterial microbiomes and results from functional experiments implicate fundamental roles of select bacterial genera from this phylum in plant protection and growth promotion. PGPB stability or persistence is an important aspect of successful PGPB application [2]. Many studies show that exogenous PGPB disappear from the resident microbial community within a few weeks after application [2]. One possible strategy to improve PGPB performance could be to select PGPB from stable host "core" microbiota.

A taxonomic analysis between duckweed-, rice-, and Arabidopsis-associated bacterial genera revealed the duckweed bacterial community matched the terrestrial leaf microbiome of rice and Arabidopsis while it was clearly distinguished from the root microbiomes of the respective terrestrial plant hosts (Fig 8A). The majority of duckweed biomass comes from its leaf-like tissue termed fronds and even though whole duckweed plants were used for analysis in this study we postulate the bacterial community of duckweed roots could be similar since both duckweed roots and fronds are in constant contact with the surrounding aquatic environment. Calculation of bacterial genera that are found in the 3 plant hosts revealed significantly less Actinobacteria taxa in the duckweed bacterial community compared to the root bacterial community (Fig 8B). Furthermore, no bacterial genera from Actinobacteria were found in the core duckweed bacterial microbiome (Fig 7) while 8 Actinobacteria genera were found in the Arabidopsis core microbiome (S12 Fig). Rice leaf tissue was also found to host less

Actinobacteria compared to Arabidopsis leaf and root tissue (Fig 8 and S11 Fig). Interestingly, Actinobacteria encompasses a distinct clade of bacteria referred to as the terrabacteria [43]. Terrabacteria evolved from a common ancestor on land and acquired traits such as a peptidoglycan layer and spore formation to help withstand stresses commonly found in terrestrial environments such as UV radiation, high salt concentrations, and drought. [ref?] Several investigations ~~into the effect of drought on the plant microbiome~~ showed an enrichment of Actinobacteria in the plant microbiome under drought conditions [44-47]. ~~One insightful report into the assembly cues of the Arabidopsis microbiome showed~~ Recruitment of Actinobacteria was Arabidopsis-specific ~~when~~ compared to controls. [ref?] Therefore, we hypothesize that terrestrial plants such as Arabidopsis may recruit Actinobacteria species into their associated bacterial communities and facilitate adaptation to stresses commonly encountered in terrestrial environments such as drought, while aquatic duckweed may not have such needs. Moreover, the similarity found between duckweed and terrestrial leaf microbiomes suggests a conserved organizational influence of plant tissue over a large evolutionary distance.

This report provides evidence that duckweed exhibits bacterial community structuring principles like those of terrestrial plants. In addition, we found that duckweed hosts a taxonomically similar bacterial community as compared to the leaf microbiome of model terrestrial plants, but with significantly fewer taxa from Actinobacteria. The taxonomic investigation of the duckweed bacterial community undertaken in this study should help inform future experimental approaches and facilitate the construction of synthetic communities from representative members of duckweed-associated bacterial genera to elucidate plant microbiota establishment principles.

Acknowledgments

Duckweed research at the Lam Lab is supported in part by a grant from the Department of Energy (DE-SC0018244) and a Hatch project (#12116) from the New Jersey Agricultural Experiment Station at Rutgers University. The Lebeis Lab acknowledges start-up funds provided to her from the University of Tennessee, Knoxville. Contribution by the Facilities Integrating Collaborations for User Science (FICUS) initiative and under Contract Nos. DE-AC02-05CH11231 (JGI) and DE-AC05-76RL01830 (EMSL) to the sequencing of the duckweed microbiome work is also gratefully acknowledged.

References

1. Glick BR. Plant growth-promoting bacteria: mechanisms and applications. Scientifica. 2012;2012.

2. Parnell JJ, Berka R, Young HA, Sturino JM, Kang Y, Barnhart DM, DiLeo MV. From the lab to the farm: an industrial perspective of plant beneficial microorganisms. Frontiers in plant science. 2016 Aug 4;7:1110.

3. Massart S, Martinez-Medina M, Jijakli MH. Biological control in the microbiome era: challenges and opportunities. Biological control. 2015 Oct 1;89:98-108.

4. Finkel OM, Castrillo G, Paredes SH, González IS, Dangl JL. Understanding and exploiting plant beneficial microbes. Current Opinion in Plant Biology. 2017 Aug 1;38:155-63.

5. Hacquard S, Garrido-Oter R, González A, Spaepen S, Ackermann G, Lebeis S, McHardy AC, Dangl JL, Knight R, Ley R, Schulze-Lefert P. Microbiota and host nutrition across plant and animal kingdoms. Cell host & microbe. 2015 May 13;17(5):603-16.

6. Bodenhausen N, Horton MW, Bergelson J. Bacterial communities associated with the leaves and the roots of *Arabidopsis thaliana*. PloS one. 2013 Feb 15;8(2):e56329.

7. Lundberg DS, Lebeis SL, Paredes SH, Yourstone S, Gehring J, Malfatti S, Tremblay J, Engelbrektson A, Kunin V, Del Rio TG, Edgar RC. Defining the core *Arabidopsis thaliana* root microbiome. Nature. 2012 Aug;488(7409):86.

8. Schlaeppi K, Dombrowski N, Oter RG, van Themaat EV, Schulze-Lefert P. Quantitative divergence of the bacterial root microbiota in *Arabidopsis thaliana* relatives. Proceedings of the National Academy of Sciences. 2014 Jan 14;111(2):585-92.

9. Edwards J, Johnson C, Santos-Medellín C, Lurie E, Podishetty NK, Bhatnagar S, Eisen JA, Sundaresan V. Structure, variation, and assembly of the root-associated microbiomes of rice. Proceedings of the National Academy of Sciences. 2015 Feb 24;112(8):E911-20.

10. Bai Y, Müller DB, Srinivas G, Garrido-Oter R, Potthoff E, Rott M, Dombrowski N, Münch PC, Spaepen S, Remus-Emsermann M, Hüttel B. Functional overlap of the *Arabidopsis* leaf and root microbiota. Nature. 2015 Dec;528(7582):364.

11. Durán P, Thiergart T, Garrido-Oter R, Agler M, Kemen E, Schulze-Lefert P, Hacquard S. Microbial interkingdom interactions in roots promote *Arabidopsis* survival. Cell. 2018 Nov 1;175(4):973-83.

12. Appenroth KJ, Borisjuk N, Lam E. Telling duckweed apart: genotyping technologies for the Lemnaceae. 应用与环境生物学报. 2013;19(1):1-0.

13. Xu J, Zhao H, Stomp AM, Cheng JJ. The production of duckweed as a source of biofuels. Biofuels. 2012 Sep 1;3(5):589-601.

14. Appenroth KJ, Sree KS, Bog M, Ecker J, Boehm V, Lorkowski S, Sommer K, Vetter W, Tolzin-Banasch K, Kirmse R, Leiterer M. Nutritional value of the duckweed species of the genus *Wolffia* (Lemnaceae) as human food. Frontiers in chemistry. 2018;6:483.

15. Michael TP, Bryant D, Gutierrez R, Borisjuk N, Chu P, Zhang H, Xia J, Zhou J, Peng H, El Baidouri M, ten Hallers B. Comprehensive definition of genome features in *Spirodela polyrhiza*

by high- depth physical mapping and short- read DNA sequencing strategies. The Plant Journal. 2017 Feb;89(3):617-35.

16. Cantó- Pastor A, Mollá- Morales A, Ernst E, Dahl W, Zhai J, Yan Y, Meyers BC, Shanklin J, Martienssen R. Efficient transformation and artificial mi RNA gene silencing in *Lemna minor*. Plant biology. 2015 Jan;17:59-65.

17. Laird RA, Barks PM. Skimming the surface: duckweed as a model system in ecology and evolution. American journal of botany. 2018 Dec 1.

18. Lam E, Appenroth KJ, Michael T, Mori K, Fakhoorian T. Duckweed in bloom: the 2nd International Conference on Duckweed Research and Applications heralds the return of a plant model for plant biology.

19. Ziegler P, Adelmann K, Zimmer S, Schmidt C, Appenroth KJ. Relative in vitro growth rates of duckweeds (Lemnaceae)–the most rapidly growing higher plants. Plant Biology. 2015 Jan;17:33-41.

20. Zhang Y, Hu Y, Yang B, Ma F, Lu P, Li L, Wan C, Rayner S, Chen S. Duckweed (*Lemna minor*) as a model plant system for the study of human microbial pathogenesis. PLoS One. 2010 Oct 25;5(10):e13527.

21. Thomson EL, Dennis JJ. Common duckweed (*Lemna minor*) is a versatile high-throughput infection model for the *Burkholderia cepacia* complex and other pathogenic bacteria. PloS one. 2013 Nov 6;8(11):e80102.

22. Wang W, Haberer G, Gundlach H, Gläßer C, Nussbaumer TC, Luo MC, Lomsadze A, Borodovsky M, Kerstetter RA, Shanklin J, Byrant DW. The *Spirodela polyrhiza* genome reveals insights into its neotenous reduction fast growth and aquatic lifestyle. Nature communications. 2014 Feb 19;5:3311.

23. Borisjuk N, Chu P, Gutierrez R, Zhang H, Acosta K, Friesen N, Sree KS, Garcia C, Appenroth KJ, Lam E. Assessment, validation and deployment strategy of a two- barcode protocol for facile genotyping of duckweed species. Plant Biology. 2015 Jan;17:42-9.

24. Lundberg DS, Yourstone S, Mieczkowski P, Jones CD, Dangl JL. Practical innovations for high-throughput amplicon sequencing. Nature methods. 2013 Oct;10(10):999.

25. Parada AE, Needham DM, Fuhrman JA. Every base matters: assessing small subunit rRNA primers for marine microbiomes with mock communities, time series and global field samples. Environmental microbiology. 2016 May;18(5):1403-14.

26. Bolyen E, Rideout JR, Dillon MR, Bokulich NA, Abnet CC, Al-Ghalith GA, Alexander H, Alm EJ, Arumugam M, Asnicar F, Bai Y. Reproducible, interactive, scalable and extensible microbiome data science using QIIME 2. Nature biotechnology. 2019 Jul 24:1.

27. Callahan BJ, McMurdie PJ, Rosen MJ, Han AW, Johnson AJ, Holmes SP. DADA2: high-resolution sample inference from Illumina amplicon data. Nature methods. 2016 Jul;13(7):581.

28. Bokulich NA, Kaehler BD, Rideout JR, Dillon M, Bolyen E, Knight R, Huttley GA, Caporaso JG. Optimizing taxonomic classification of marker-gene amplicon sequences with QIIME 2's q2-feature-classifier plugin. Microbiome. 2018 Dec;6(1):90.

29. Camacho C, Coulouris G, Avagyan V, Ma N, Papadopoulos J, Bealer K, Madden TL. BLAST+: architecture and applications. BMC bioinformatics. 2009 Dec;10(1):421.

30. Katoh K, Misawa K, Kuma KI, Miyata T. MAFFT: a novel method for rapid multiple sequence alignment based on fast Fourier transform. Nucleic acids research. 2002 Jul 15;30(14):3059-66.

31. Price MN, Dehal PS, Arkin AP. FastTree 2–approximately maximum-likelihood trees for large alignments. PloS one. 2010 Mar 10;5(3):e9490.

32. Faith DP. Conservation evaluation and phylogenetic diversity. Biological conservation. 1992 Jan 1;61(1):1-0.

33. Chen J, Bittinger K, Charlson ES, Hoffmann C, Lewis J, Wu GD, Collman RG, Bushman FD, Li H. Associating microbiome composition with environmental covariates using generalized UniFrac distances. Bioinformatics. 2012 Jun 17;28(16):2106-13.

34. Fernandes AD, Reid JN, Macklaim JM, McMurrough TA, Edgell DR, Gloor GB. Unifying the analysis of high-throughput sequencing datasets: characterizing RNA-seq, 16S rRNA gene

sequencing and selective growth experiments by compositional data analysis. Microbiome. 2014 Dec;2(1):15.

35. Gloor GB, Macklaim JM, Pawlowsky-Glahn V, Egozcue JJ. Microbiome datasets are compositional: and this is not optional. Frontiers in microbiology. 2017 Nov 15;8:2224.

36. Thorsen J, Brejnrod A, Mortensen M, Rasmussen MA, Stokholm J, Al-Soud WA, Sørensen S, Bisgaard H, Waage J. Large-scale benchmarking reveals false discoveries and count transformation sensitivity in 16S rRNA gene amplicon data analysis methods used in microbiome studies. Microbiome. 2016 Dec;4(1):62.

37. Robbins C, Thiergart T, Hacquard S, Garrido-Oter R, Gans W, Peiter E, Schulze-Lefert P, Spaepen S. Root-associated bacterial and fungal community profiles of *Arabidopsis thaliana* are robust across contrasting soil P levels. Phytobiomes. 2018 Jan 30;2(1):24-34.

38. Agler MT, Ruhe J, Kroll S, Morhenn C, Kim ST, Weigel D, Kemen EM. Microbial hub taxa link host and abiotic factors to plant microbiome variation. PLoS Biology. 2016 Jan 20;14(1):e1002352.

39. Thiergart T, Duran P, Ellis T, Garrido-Oter R, Kemen E, Roux F, Alonso-Blanco C, Agren J, Schulze-Lefert P, Hacquard S. Root microbiota assembly and adaptive differentiation among European *Arabidopsis* populations. bioRxiv. 2019 Jan 1:640623.

40. Vannier N, Agler M, Hacquard S. Microbiota-mediated disease resistance in plants. PLoS pathogens. 2019 Jun 13;15(6):e1007740.

41. Toyama T, Kuroda M, Ogata Y, Hachiya Y, Quach A, Tokura K, Tanaka Y, Mori K, Morikawa M, Ike M. Enhanced biomass production of duckweeds by inoculating a plant growth-promoting bacterium, *Acinetobacter calcoaceticus* P23, in sterile medium and non-sterile environmental waters. Water Science and Technology. 2017 Sep 27;76(6):1418-28.

42. Yamaga F, Washio K, Morikawa M. Sustainable biodegradation of phenol by *Acinetobacter calcoaceticus* P23 isolated from the rhizosphere of duckweed *Lemna aoukikusa*. Environmental science & technology. 2010 Jul 21;44(16):6470-4.

43. Battistuzzi FU, Hedges SB. A major clade of prokaryotes with ancient adaptations to life on land. Molecular biology and evolution. 2008 Nov 6;26(2):335-43.

44. Santos-Medellín C, Edwards J, Liechty Z, Nguyen B, Sundaresan V. Drought stress results in a compartment-specific restructuring of the rice root-associated microbiomes. MBio. 2017 Sep 6;8(4):e00764-17.

45. Naylor D, DeGraaf S, Purdom E, Coleman-Derr D. Drought and host selection influence bacterial community dynamics in the grass root microbiome. The ISME journal. 2017 Dec;11(12):2691.

46. Xu L, Naylor D, Dong Z, Simmons T, Pierroz G, Hixson KK, Kim YM, Zink EM, Engbrecht KM, Wang Y, Gao C. Drought delays development of the sorghum root microbiome and enriches for monoderm bacteria. Proceedings of the National Academy of Sciences. 2018 May 1;115(18):E4284-93.

47. Fitzpatrick CR, Copeland J, Wang PW, Guttman DS, Kotanen PM, Johnson MT. Assembly and ecological function of the root microbiome across angiosperm plant species. Proceedings of the National Academy of Sciences. 2018 Feb 6;115(6):E1157-65.

Supporting Information

S1 Fig. Testing of QIIME 2 feature-classifier with different classification methods. Different methods for the q2 feature-classifier plugin were tested for their ability to classify ASVs at the phylum level. The effect of using different databases was tested by either using the Greengenes 13_8 99 % OTUs reference database (gg) or SILVA 132 (silva) database. dada2 = reads remaining after q2-dada2 quality control, NB = q2 feature classifier using naive bayes method, blast = q2 feature classifier using BLAST+ consensus method, vsearch = q2 feature-classifier using VSEARCH consensus method

S2 Fig. Rarefaction analysis. (A) The number of ASVs observed in duckweed and ambient water samples from Caldwell House and Passion Puddle at different sampling depths. The solid line intercepting the x-axis represents a sampling depth of 3664 reads. (B) The number of ASVs observed in Princeton Meadows year 1 samples at different sampling depths. Samples were rarefied to 112500 reads. (C) The number of ASVs observed in Princeton Meadows year 2 samples at different sampling depths. Samples were rarefied to 108000 reads.

S3 Fig. Duckweed hosts a less diverse bacterial community than the surrounding water that is phylogenetically similar between different locations. (A) The total number of ASVs observed and assessment of the phylogenetic diversity using Faith's PD index for duckweed and ambient water samples collected from both Caldwell House and Passion Puddle sites. Wilcoxon rank sum test was used for comparison of number of ASVs and Faith's PD index (p-value < 0.05 = "*", p-value < 0.01 = "**", p-value < 0.001 = "***", p-value < 0.0001 = "****"). (B) Principle coordinate analysis of duckweed and ambient water samples from Caldwell House and Passion Puddle sites using unweighted UniFrac and generalized UniFrac distances.

S4 Fig. Number of plastid and bacteria reads normalized to total reads in initial quasi-gnotobiotic duckweed tissue compared to duckweed tissue several days after inoculation with wastewater from both Princeton Meadows year 1 and year 2 studies. Pairwise comparison was performed using Wilcoxon rank sum test (p-values are displayed). Initial = initial starting duckweed tissue, ww_inoculated = duckweed tissue inoculated with Princeton Meadows wastewater

S5 Fig.

S5 Fig. Analysis of bacterial ASVs for the Princeton Meadows Year 1 study. (A) Shared and specific bacterial ASVs between initial Sp9509 tissue (Initial Sp9509 = Sp9509 tissue at 0

days), initial wastewater inoculum (initial WW), and Sp9509 inoculated with wastewater (WW-Inoculated Sp9509). (B) Abundance of stable ASVs (median CLR > 0) found within initial Sp9509 and all other ASVs within the same bacterial genera found in wastewater-inoculated Sp9509. Bacterial ASVs found in initial Sp9509 are colored purple while bacterial ASVs in wastewater-inoculated Sp9509 are colored blue (C) Bacterial ASVs found in wastewater-inoculated Sp9509 (n = 16) were analyzed. Each data point in the graph represents a bacterial ASV. These bacterial ASVs were grouped by origin and were either specific to wastewater-inoculated Sp9509 (**post)**, found in both wastewater-inoculated Sp9509 and starting Sp9509 tissue **(post_t0)**, found in both wastewater-inoculated Sp9509 and initial wastewater inoculum **(post_ww)**, or found in wastewater-inoculated Sp9509, initial wastewater inoculum, and starting Sp9509 tissue **(post_ww_t0)**. These ASVs were also categorized by the number of wastewater-inoculated Sp9509 samples they were observed in as follows: **1 sample** = found in only 1 wastewater-inoculated Sp9509 sample, **< 25 %** = found in greater than 1 % but less than 25 % of wastewater-inoculated Sp9509 samples, **< 50 %** = found between 25 % and less than 50 % of wastewater-inoculated Sp9509 samples, **< 75 %** = found between 50 % and less than 75 % of wastewater-inoculated Sp9509 samples, **< 100 %** = found between 75 % and less than 100 % of wastewater-inoculated Sp9509 samples, **16 samples** = found in all 16 wastewater-inoculated Sp9509 samples. The number of bacterial ASVs observed in each prevalence category are displayed on top. The abundance of each ASV was measured by calculating the median log2 centered-log ratio (median CLR). CLR values close to 0 represent an average abundance in samples, values less than 0 signify a less than average abundance in samples, and values greater than 0 signify a higher than average abundance in samples.

S6 Fig. Taxonomic analysis of the Princeton Meadows year 1 study at the phylum, family, and genus level. Relative abundance of duckweed-associated bacteria at A) phylum and B) family levels. C) Genus level analysis showing the number of genera shared between sample

types, between different time points, and between DAB1A-inoculated and un-inoculated samples.

S7 Fig. Analysis of bacterial ASVs found in starting duckweed tissue used for the Princeton Meadows year 2 study. (A) Unique and shared bacterial ASVs between initial starting duckweed (Initial DK) and Princeton Meadows year 2 wastewater-inoculated duckweed (Post DK) tissues. (B) Abundance of bacterial ASVs found in both starting tissue and wastewater-inoculated duckweed across different time points. Reads were log2 centered-log ratio (clr) transformed with a prior of 0.001 and the median clr was quantified at different time points for Lm5576 and Sp9509.

S8 Fig. Taxonomic analysis of bacterial community in Princeton Meadows year 2 study at phylum, family and genus level. Relative abundance of duckweed-associated bacteria at A) phylum and B) family level for each sample. C) Genus level analysis showing shared and unique bacterial genera between duckweed species, between duckweed samples at 2 and 7 days, and between water treated and salt and detergent treated duckweed samples. "0" = 0 day samples; "2" = 2 day samples; "7" = 7 day samples; "W" = duckweed samples treated with water; "SD" = duckweed samples treated with salt and detergent; "Sp9509" = *S. polyrhiza* 9509 samples; "Lm5576" = *L. minor* 5576 samples

S9 Fig. Different duckweed species host a similar abundance of bacterial ASVs. PCoA using Bray-Curtis distance metric calculated from abundance of bacterial ASVs in Princeton Meadows year 2 study.

S10 Fig. Core bacteria abundance varies between different studies. The log2 centered-log ratio (clr) using a prior of 0.001 was computed for core bacteria across different duckweed studies. Kruskal-Wallis test p-values are displayed.

S11 Fig. Conservation of rice-associated bacteria. The percentage of samples each bacteria was observed in for each location was calculated. Core bacteria represent bacteria that were observed in all locations. The color black illustrated in heatmaps means bacterial genus was not observed in any samples for that location.

S12 Fig. Conservation of Arabidopsis-associated bacteria. The percentage of samples each bacteria was observed in for each location was calculated. Core bacteria represent bacteria that were observed in all locations. The color black illustrated in heatmaps means bacterial genus was not observed in any samples for that location.

S1 File. Sample metadata and library Information. Excel spreadsheet containing metadata and library processing stats for samples from (A) Caldwell House and Passion Puddle, (B) Princeton Meadows year 1, and (C) Princeton Meadows year 2 studies.

S2 File. Taxonomic analysis at phylum, family, and genus level for Caldwell House and Passion Puddle. Excel workbook containing relative abundances of duckweed-associated bacteria at the (A) phylum and (B) family level. Information on duckweed genera (C-D), tissue-specific (E-F), and location-specific (G-H) bacterial genera is also included. RA = relative abundance, Sample_Percent = percentage of samples genus was found in, OTU_Count = number of ASVs within bacterial genus, Mean_RA_Rank = bacterial genera were ranked by their mean relative abundance, sample_type = indicates whether genus was observed in ambient water (AW) and/or duckweed (DK)

S3 File. Differential enrichment of bacteria between duckweed and ambient water from Caldwell House and Passion Puddle using ALDEx2. ALDEx2 analysis of (A) Caldwell House and (B) Passion Puddle samples. rab.win.ambient_water = median clr value in ambient water samples, rab.win.rinsed_tissue = median clr in duckweed samples, diff.btw = median difference in clr values between duckweed and ambient water samples, diff.win = median of largest difference in clr values within duckweed and ambient water samples, effect = median effect size (diff.btw / diff.win), we.eBH = expected benjamini-hochberg correct p-value of Welch's t-test, wi.eBH = expected benjamini-hochberg correct p-value of Wilcoxon rank test

S4 File. Analysis of bacterial ASVs found in duckweed for the Princeton Meadows Year 1 study. (A) Number of reads for each bacterial ASV found within initial Sp9509 tissue. (B) Reads belonging to the 35 ASVs found in both initial Sp9509 and wastewater-inoculated Sp9509 were centered-log ratio transformed with a prior of 0.001 to quantify ASV abundance at different time points. (C) Information for bacterial ASVs found in wastewater-inoculated Sp9509 samples. ASV type indicates whether bacterial ASV was either specific to wastewater-inoculated Sp9509 (post), found in both wastewater-inoculated Sp9509 and starting Sp9509 tissue (post_t0), found in both wastewater-inoculated Sp9509 and initial wastewater inoculum (post_ww), or found in wastewater-inoculated Sp9509, initial wastewater inoculum, and starting Sp9509 tissue (post_ww_t0). ASV prevalence indicates the quantity of wastewater-inoculated Sp9509 samples the bacterial ASV was observed in: **1 sample** = found in only 1 wastewater-inoculated Sp9509 sample, **< 25 %** = found in greater than 1 % but less than 25 % of wastewater-inoculated Sp9509 samples, **< 50 %** = found between 25 % and less than 50 % of wastewater-inoculated Sp9509 samples, **< 75 %** = found between 50 % and less than 75 % of wastewater-inoculated Sp9509 samples, **< 100 %** = found between 75 % and less than 100 % of wastewater-inoculated Sp9509 samples, **100 %** = found in all 16 wastewater-inoculated Sp9509 samples.

To quantify bacterial ASV abundance the median log2 centered-log ratio was calculated (median clr). CLR values close to 0 represent an average abundance, values less than 0 signify a less than average abundance, and values greater than 0 signify a higher than average abundance in wastewater-inoculated Sp9509 samples. feature_id = alias for ASV

S5 File. Relative abundances of duckweed-associated bacteria at phylum and family level and genus level information from Princeton Meadows year 1 study. Relative abundance at (A) phylum and (B) family levels. Information for bacterial genera (C) specific to Sp9509 tissue compared to wastewater and ambient wastewater, (D) 5 day specific Sp9509-associated bacterial genera, and (E) 10 day specific Sp9509-associated bacterial genera. RA = relative abundance, Sample_Percent = percentage of samples bacterial genus was found in, OTU_Count = number of ASVs from bacterial genus, Mean_RA_Rank = rank was assigned according to mean relative abundance

S6 File. Differential abundance testing between sample types from Princeton Meadows year 1 using ALDEx2. (A) Kruskal-Wallis and generalized linear model construction for differential abundance testing at bacterial genus level between sample types. (B) Pairwise comparison between wastewater and ambient wastewater samples for differential enrichment. Genera with effect sizes less than -1 are more abundant in ambient wastewater than wastewater. (C) Pairwise comparison between wastewater and Sp9509 tissue samples. Genera enriched on Sp9509 compared to wastewater have effect sizes less than -1. (D) Pairwise comparison between Sp9509 and ambient wastewater samples. Genera enriched on duckweed compared to ambient wastewater have effect sizes greater than 1. rab.win.ambient_water = median clr value in ambient wastewater samples, rab.win.wastewater = median clr value in wastewater samples, rab.win.treated_tissue = median clr in duckweed samples, we.eBH = expected benjamini-hochberg correct p-value of Welch’s t-test, diff.btw = median difference in

clr values between sample type, diff.win = median of largest difference in clr values within sample type, effect = median effect size (diff.btw / diff.win)

S7 File. Differential abundance testing between different time points from Princeton Meadows year 1 using ALDEx2. Pairwise comparison between 5 day and 10 day (A) ambient wastewater and (B) Sp9509 samples.

S8 File. Analysis of bacterial ASVs found in initial duckweed tissue used for the Princeton Meadows Year 2 study. (A) Number of reads for each bacterial ASV found in starting duckweed tissue. (B) Abundance of bacterial ASVs found in both starting tissue and wastewater-inoculated duckweed across different time points. Reads were log2 centered-log ratio (clr) transformed with a prior of 0.001 and the median clr was quantified at different time points for Lm5576 and Sp9509.

S9 File. Differential abundance testing between different factors including species, time, and treatment. ALDEx2 was used to generate a (A) generalized linear model and perform pairwise comparisons to determine the effect of duckweed species, time, and sample treatment on community composition. (B) Pairwise comparison between Lm5576 and Sp9509 for changes in duckweed-associated bacteria abundance. (C) Pairwise comparison of bacterial abundance between 2 day and 7 day duckweed samples. (D) Pairwise comparison of bacterial abundance between water treated and salt and detergent treated duckweed samples. Initial duckweed samples (t = 0 days) were excluded from testing. rab.win.L_minor370_DWC112 = median clr value in Lm5576 samples, rab.win.S_polyrhiza432_9509 = median clr value in Sp9509 samples, rab.win.2 = median clr value in 2 day duckweed samples, rab.win.7 = median clr value in 7 day duckweed samples, rab.win.water_treated = median clr value in water treated duckweed samples, rab.win.SD_treated = median clr value in salt and detergent treated

duckweed samples, we.eBH = expected benjamini-hochberg correct p-value of Welch's t-test, diff.btw = median difference in clr values between factors tested, diff.win = median of largest difference in clr values within factors tested, effect = median effect size (diff.btw / diff.win)

S10 File. Genus level information for duckweed-associated bacteria from Princeton Meadows year 2 study. Information for bacterial genera specific to (A) Lm5576 duckweed samples, (B) specific to *S. polyrhiza* duckweed samples, C) 2 day specific duckweed-associated bacteria, D) 7 day specific duckweed-associated bacterial genera, E) bacterial genera specific to water treated duckweed samples, F) bacteria specific to SD treated duckweed samples, G) bacteria specific to duckweed samples not pre-inoculated with any bacteria, H) bacteria specific to duckweed samples pre-inoculated with DAB 1A, I) bacteria specific to duckweed samples pre-inoculated with DAB 3D, and J) bacteria specific to duckweed samples pre-inoculated with DAB 5A.

S11 File. Metadata for comparative analysis of duckweed-, rice-, and Arabidopsis-associated bacterial communities.

S1 Table. Pairwise comparison showing the total number of different bacterial genera associated with duckweed between locations. Bacterial genera that were present in only 1 sample from all study samples were filtered out.

S1 Protocol. Collecting duckweed and ambient water samples from the environment.

S2 Protocol. Co-isolation of duckweed and bacteria nucleic acid.

S3 Protocol. PCR to detect bacteria associated with duckweed.

 Click here to access/download;Figure;Fig_1.tif

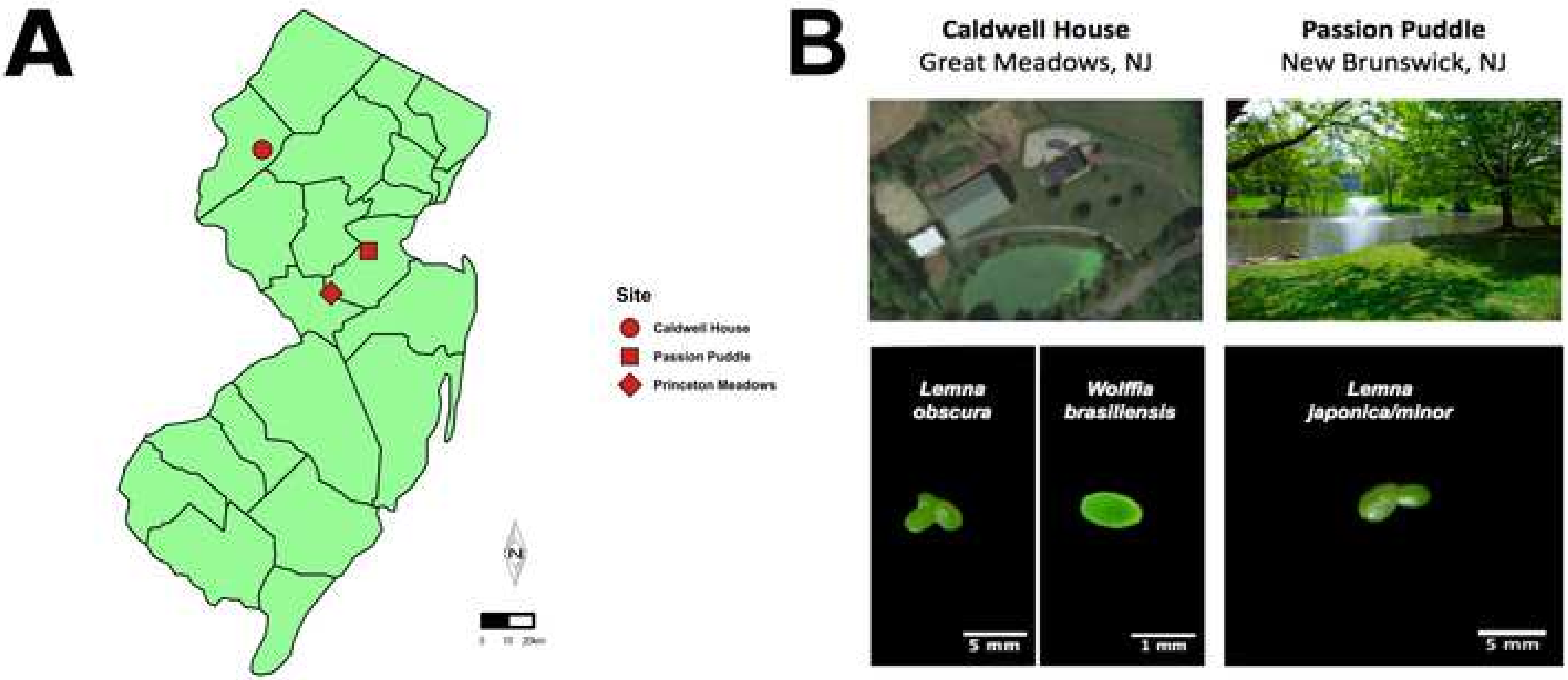

Figure

Click here to access/download;Figure;Fig_2.tif

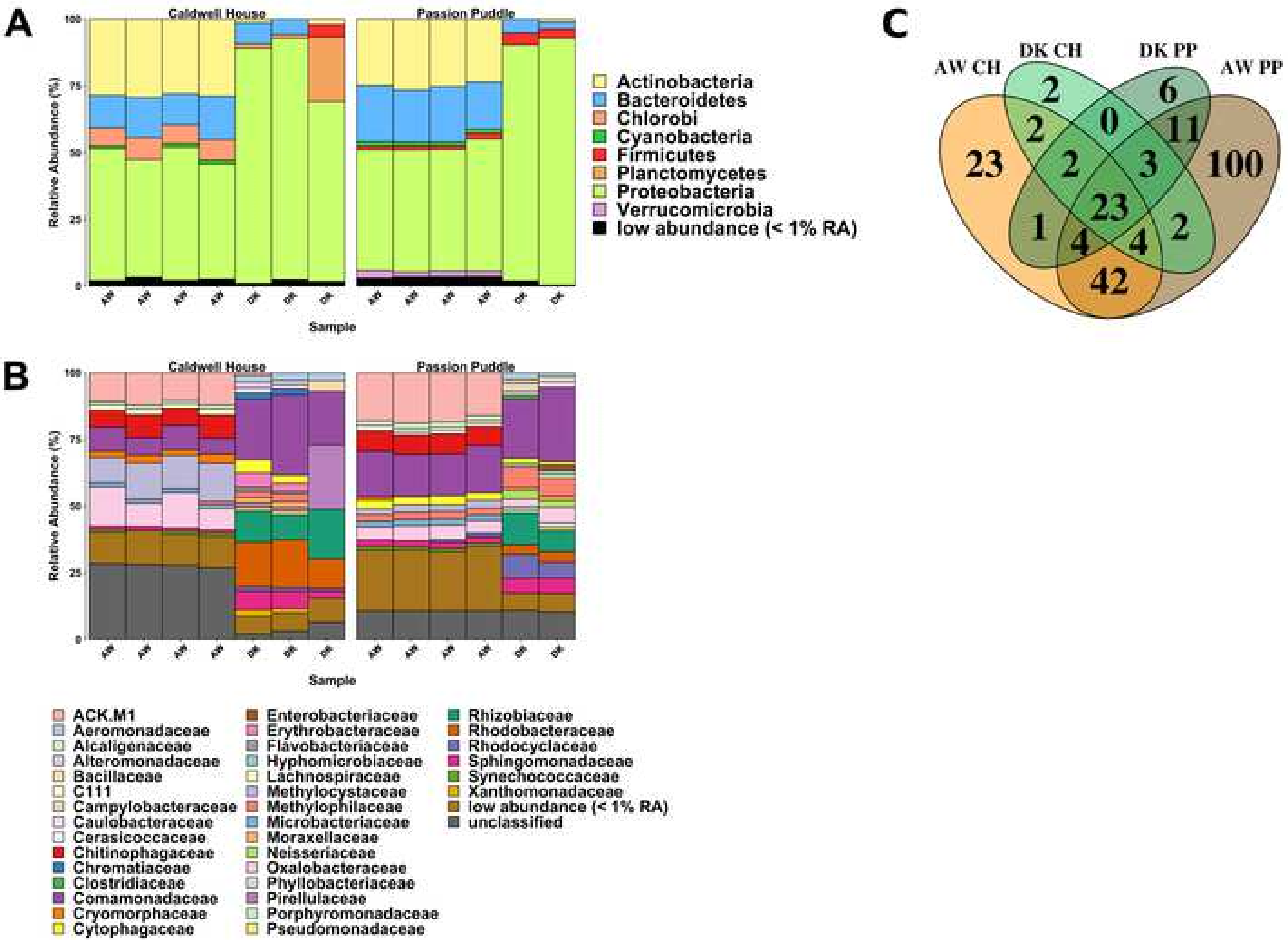

Figure

Click here to access/download;Figure;Fig_3.tif

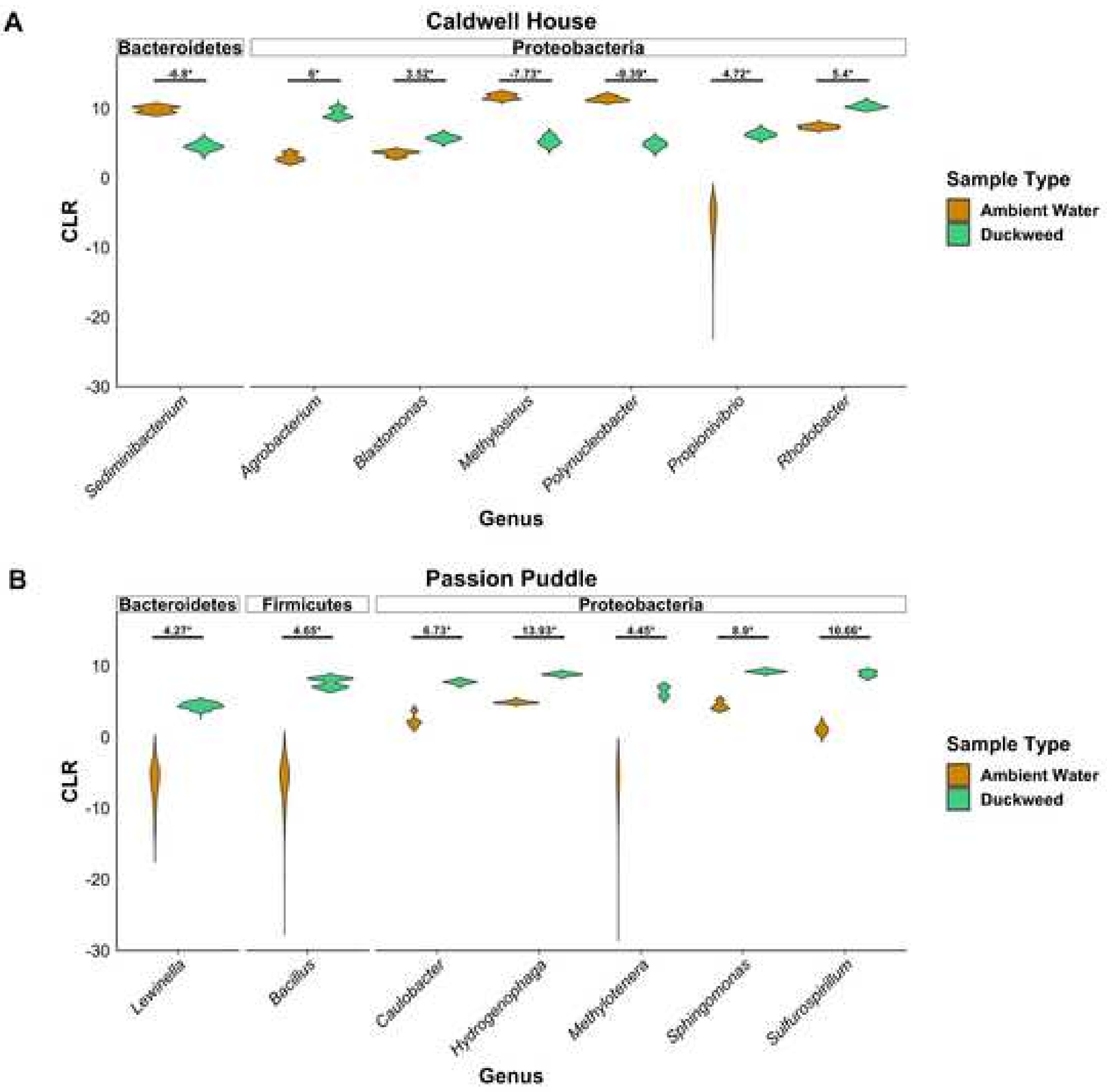

Figure

Click here to access/download;Figure;Fig_4.tif

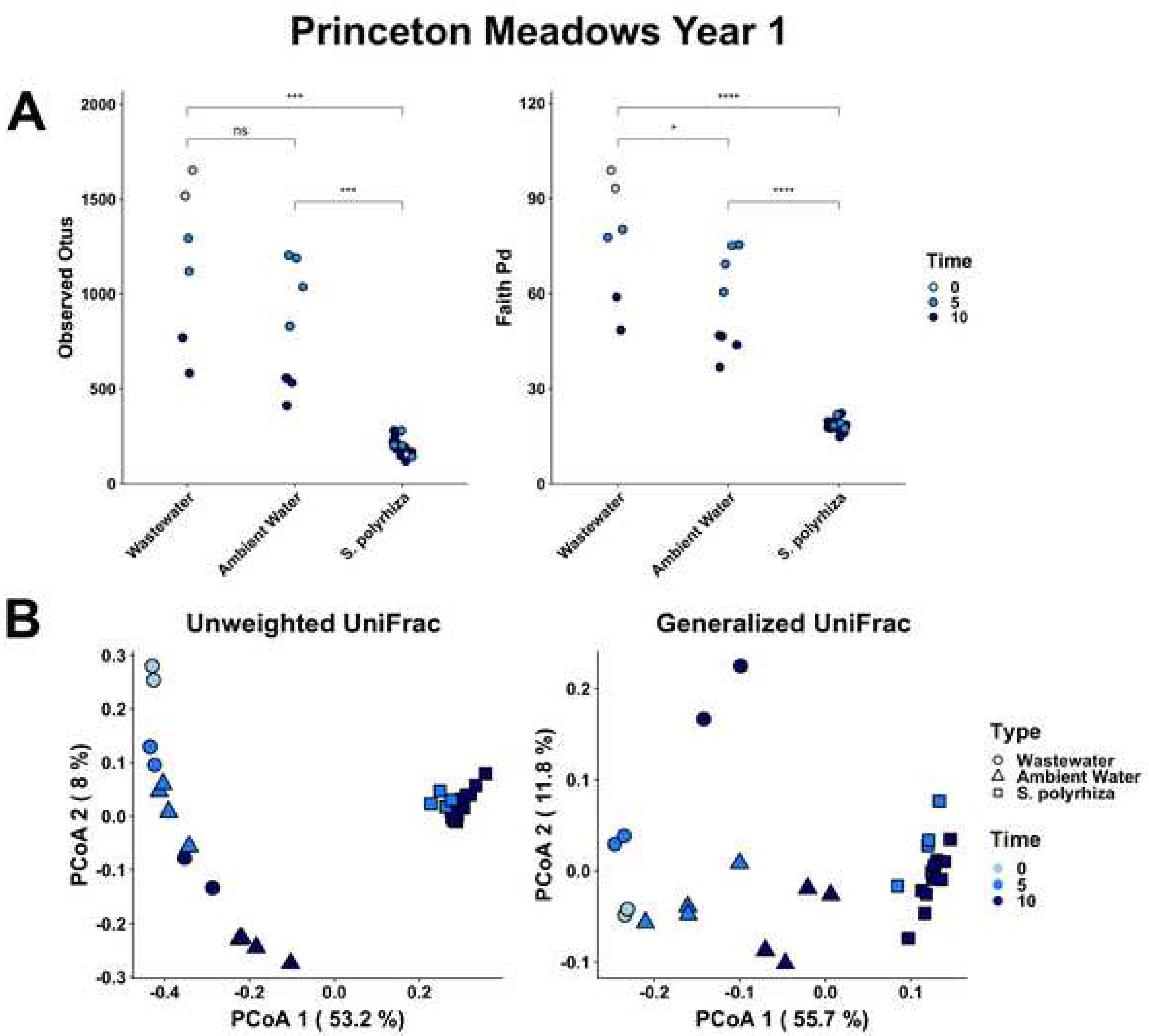

Figure

Click here to access/download;Figure;Fig_5.tif

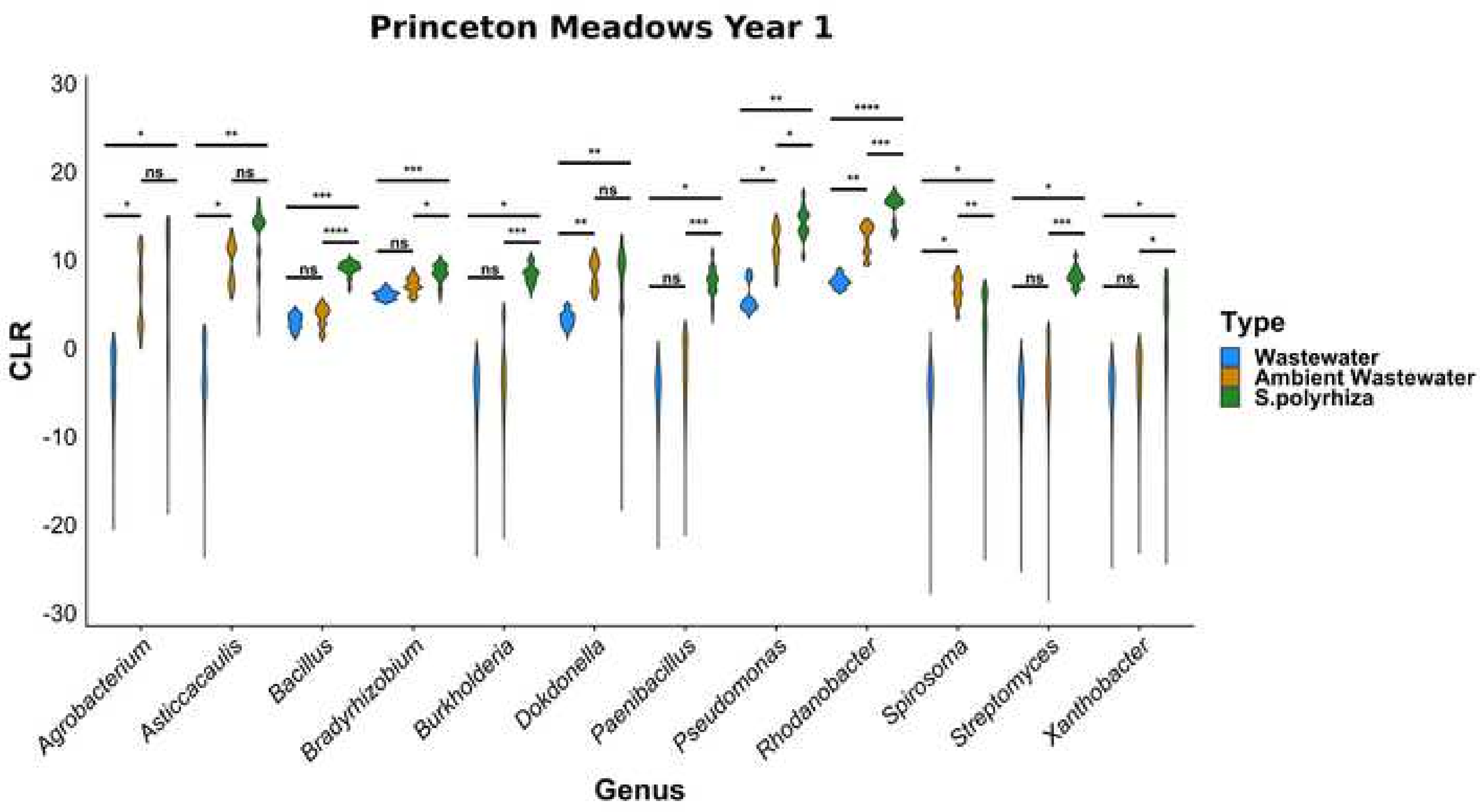

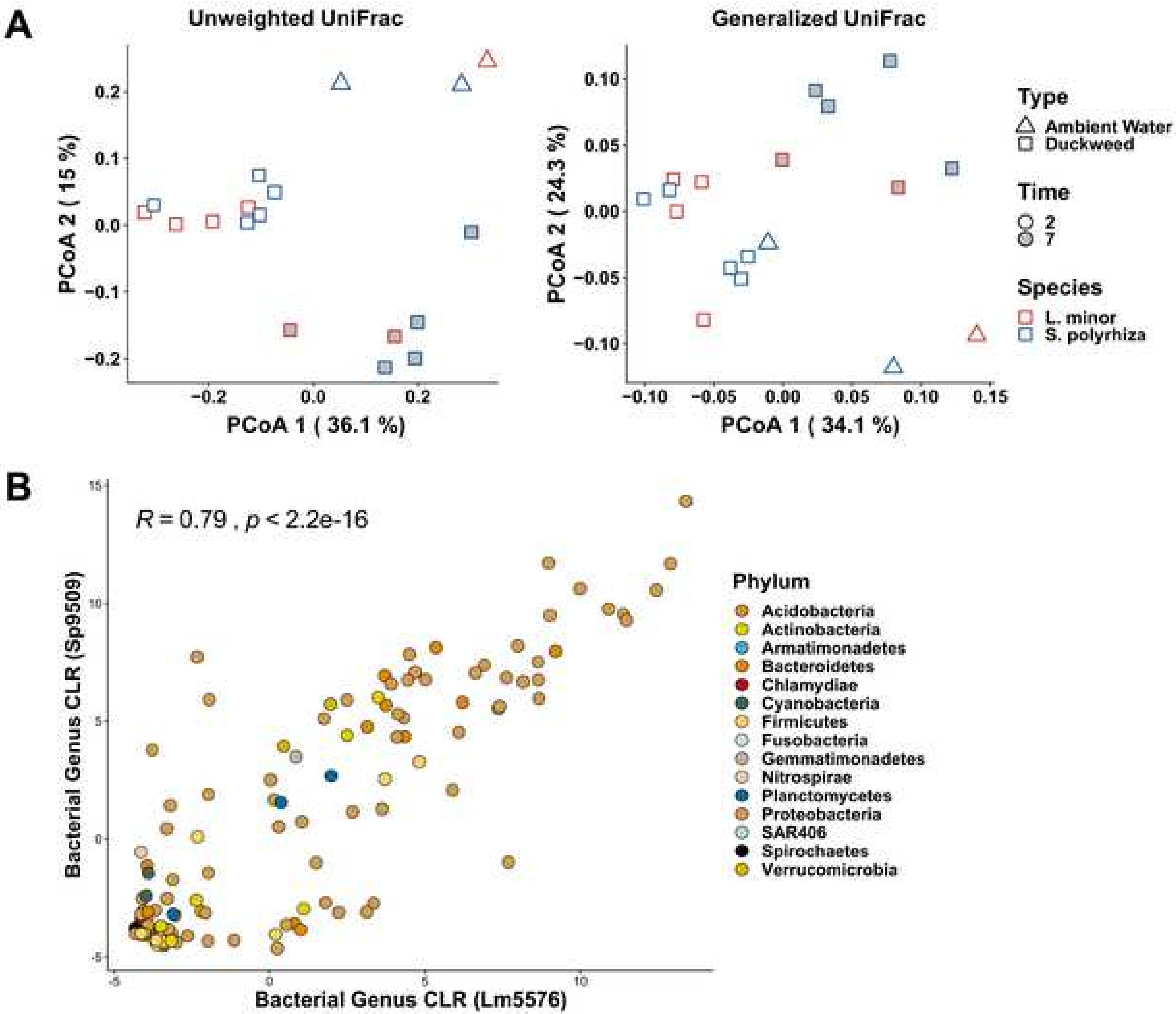
A
Unweighted UniFrac
Generalized UniFrac
PCoA 2 (15 %)
PCoA 1 (36.1 %)
PCoA 2 (24.3 %)
PCoA 1 (34.1 %)
Type
Ambient Water
Duckweed
Time
2
7
Species
L. minor
S. polyrhiza
B
R = 0.79 , p < 2.2e-16
Bacterial Genus CLR (Sp9509)
Bacterial Genus CLR (Lm5576)
Phylum
Acidobacteria
Actinobacteria
Armatimonadetes
Bacteroidetes
Chlamydiae
Cyanobacteria
Firmicutes
Fusobacteria
Gemmatimonadetes
Nitrospirae
Planctomycetes
Proteobacteria
SAR406
Spirochaetes
Verrucomicrobia

Figure

Click here to access/download;Figure;Fig_7.tif

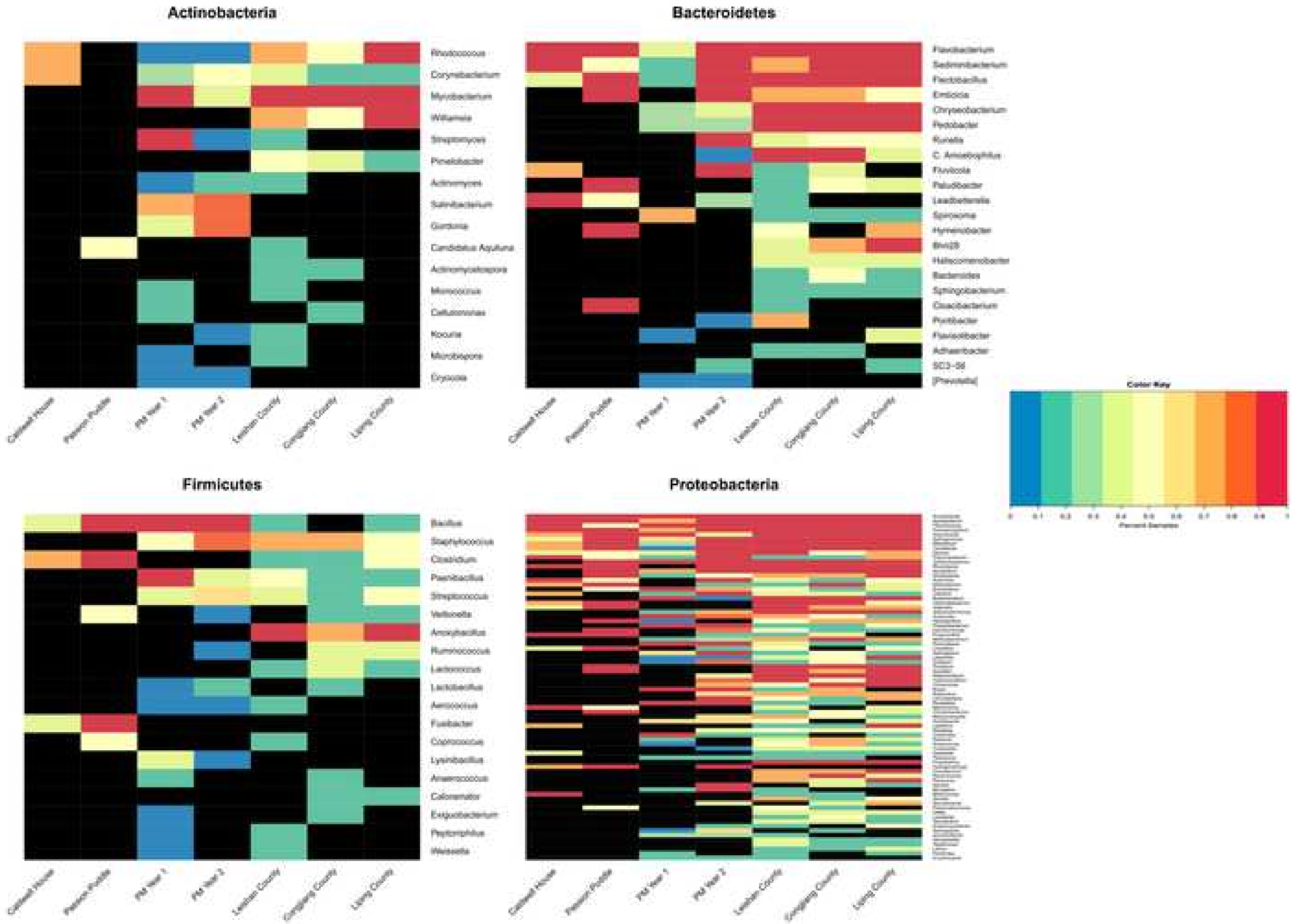

Figure

Click here to access/download;Figure;Fig_8.tif

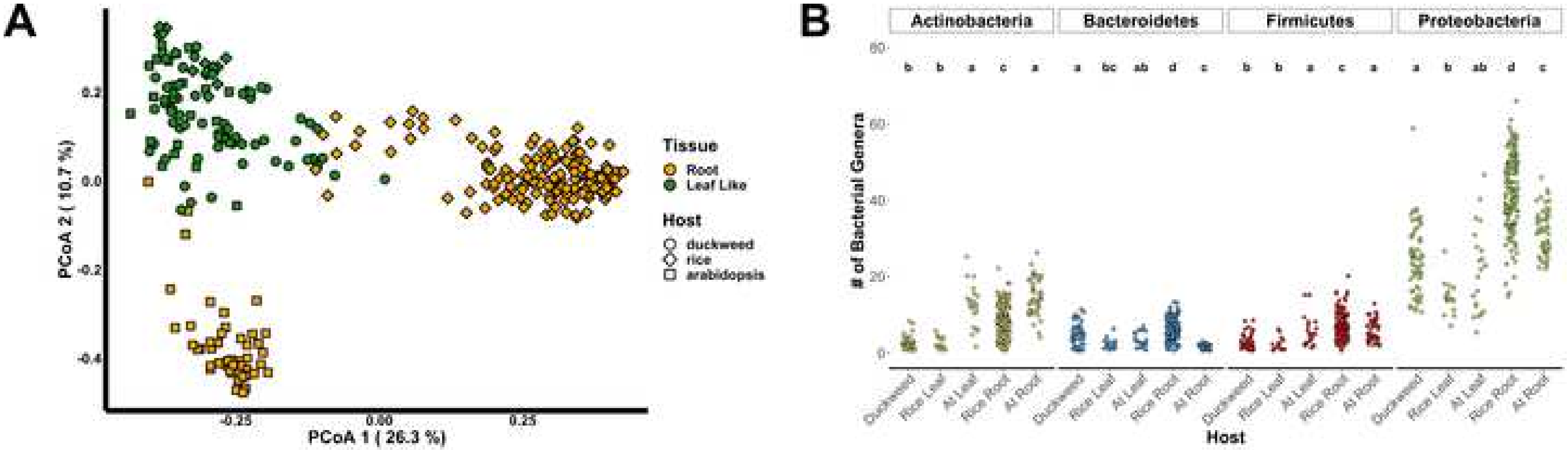

Click here to access/download
Supporting Information - Compressed/ZIP File Archive
2019-PLos_One-SI.zip
